# Factors Influencing the Satisfaction of Second Language Learners of Chinese in Online Courses

**DOI:** 10.3390/bs14050387

**Published:** 2024-05-04

**Authors:** Xingrong Guo, Xiang Li, Yiming Guo

**Affiliations:** 1 College of Foreign Languages, Shanghai Maritime University, 1550 Haigang Ave, Shanghai 201306, China; hiiragilee@163.com; 2 School of Economics and Management, Shanghai Maritime University, 1550 Haigang Ave, Shanghai 201306, China; ymguo@shmtu.edu.cn

**Keywords:** online teaching, second language learners of Chinese, learners’ satisfaction, influencing factors

## Abstract

The study aims to investigate the relationship among the key factors affecting second language learners’ satisfaction with online Chinese courses and their willingness to continue utilizing them by constructing a Model of Chinese Learners’ Satisfaction in Online Courses. Additionally, the influence of participants’ individual differences was also explored. A total of 203 second language learners of Chinese participated in the questionnaire survey, with 5 learners further participating in interviews. Learner expectations, learner perceived quality, and learner perceived value were identified as important factors influencing learner satisfaction and willingness to continue using the online course. The results of the questionnaire survey showed that (1) learner individual differences, such as age, Chinese proficiency, weekly study duration, and offline Chinese course experience, significantly influence learner satisfaction. (2) Learner expectations have a significant positive impact on perceived quality, while perceived quality positively affects perceived value. (3) Learner satisfaction significantly influences the willingness to continue using online courses. (4) The results of the interview revealed that most learners still prefer traditional offline courses, indicating that online teaching has several shortcomings and deficiencies. Overall, this study provides some scientific and reasonable decision-making references for improving online teaching methods, aiming to enhance learner satisfaction and promote the development of online education.

## 1. Introduction

In recent decades, online learning has emerged as a prominent topic of interest in the field of education due to its inherent advantages, offering learners the flexibility to overcome constraints of time, geographic location, and other factors [1]. In particular, online education has never gained as much prominence or global attention as it did in 2020, primarily due to the impact of the COVID-19 pandemic [2]. The sudden outbreak caught institutions off guard, leaving them with little preparation, but nevertheless, they were compelled to swiftly transition to online education [3,4]. In response to the global outbreak of the COVID-19 pandemic, educational policies such as “Suspending Classes Without Stopping Learning” were implemented in China [5], and virtually almost all educational institutions worldwide integrated online learning into various subject teaching, making it the most effective method for emergency education [6]. Aligning with this trend, a growing variety of online teaching methods, approaches, and platforms have been employed to ease the transition to online education.

Numerous scholarly investigations have underscored the potential of online learning to furnish learners with a plethora of favorable educational experiences [7,8]. This propensity culminates in heightened learner contentment when contrasted with the conventional face-to-face instructional mode [9]. Baharum et al. [10] found that most participants expressed a preference for traditional face-to-face learning. Issues such as lack of course guidance and poor internet connectivity were cited as contributing factors to diminished engagement and satisfaction. Among the plausible factors contributing to diminished engagement and satisfaction, optimal course design and pedagogical strategies within the domain of online instruction merit consideration [11]. The multifarious array of scholarly perspectives necessitates a more exhaustive, methodical, and profound exploration into the realm of online learning satisfaction, a pivotal endeavor aimed at fortifying the quality of service provision in online learning curricula and honing the evaluative framework for the caliber of online pedagogy.

Student satisfaction plays a pivotal role in assessing the effectiveness and quality of educational programs [12]. The satisfaction of online learners significantly influences the efficacy of online courses and contributes to enhancing the overall quality of online learning experiences [13]. As student satisfaction increases, so does their inclination to persist in using online learning platforms [14]. Some researchers have investigated the satisfaction of second language learners in synchronous online learning environments and emphasized the importance of this topic for optimizing teaching [15]. Some research has indicated that studying the satisfaction of international students with online Chinese learning provides practical significance for the sustainable development of online Chinese education in China and other regions [16]. Previous studies have predominantly focused on general online education or languages other than Chinese [15], necessitating dedicated research into the distinct factors affecting learner satisfaction in online Chinese language courses. Given the significance of student satisfaction in educational programs and the impact it has on online learning efficacy, it is imperative to delve deeper into understanding learner satisfaction within specific contexts such as online Chinese language courses for second language learners.

Through literature review, it was found that most studies on online learning satisfaction have focused on students’ learning experiences (e.g., [17,18]). Only a few studies have investigated students’ satisfaction with online language learning [15]. Many of these have been concentrated on the online learning of English as a foreign language [15]. There is limited research exploring the satisfaction of international students in China with online Chinese language learning [16]. There are even fewer studies on the factors influencing the satisfaction of Chinese language learners with online teaching.

Chinese, as one of the most important languages in the world, holds a significant position in international communication and business activities. Therefore, understanding the satisfaction of second language learners of Chinese with online courses helps educators better comprehend their learning needs and challenges, thereby providing more effective teaching and support. In fact, against the backdrop of the increasing prevalence of online education, online courses for second language learners of Chinese have emerged as a popular avenue for language acquisition. However, it remains unclear how individual differences impact learner satisfaction with online Chinese courses, what factors specifically influence learner satisfaction in this context, and how learner satisfaction with online Chinese courses affects their intention to continue using them.

This study aims to investigate the causal relationships among variables influencing learners’ online satisfaction and their willingness to continue learning online courses by constructing a Model of Chinese Learners’ Satisfaction in Online Courses (MCLSOC). The primary goal is to explore the relationship among influencing factors such as learner expectations, perceived quality, perceived value, satisfaction, and willingness to continue using online courses. The specific objectives are listed as follows:

Objective 1: To examine the impact of individual differences on the factors influencing learner satisfaction and their willingness to continue learning online Chinese courses.

Objective 2: To explore the key factors influencing students’ satisfaction and their relationship.

Objective 3: To investigate how learner satisfaction with online Chinese courses affects their intention to continue using them.

Moreover, the study proposes optimal strategies for enhancing online education satisfaction to better meet student learning needs and promote the development of online Chinese language teaching.

The research questions were as follows:

Research question 1 (RQ1). How do individual differences impact the main factors that influence learners’ satisfaction with online Chinese courses?

Research question 2 (RQ2). What are the main factors influencing students’ satisfaction and their willingness to continue learning online Chinese courses?

Research question 3 (RQ3). How does learner satisfaction with online Chinese courses affect their intention to continue using them?

The remainder of the paper is organized as follows: Section 2 encompasses a literature review and theoretical framework, detailing a review of relevant literature on learner satisfaction in online education and proposing a theoretical framework foundation. Section 3 outlines the methodology employed, including the design of the questionnaire and interview survey. Section 4 presents the empirical findings of the proposed satisfaction model for online Chinese language learners, including reliability and validity tests, statistical analysis of differences, correlation analysis, regression analysis, and interview survey results, highlighting the factors that influence learner satisfaction with online courses. Section 5 discusses the implications of the findings and offers recommendations for improving learners’ online satisfaction. Finally, the conclusion summarizes the key findings and proposes improvement strategies while also acknowledging limitations and suggesting areas for further research.

## 2. Literature Review and Theoretical Framework

### 2.1. Literature Review

Online teaching has yet to be uniformly defined. Terms such as online teaching, network teaching, e-learning, distance teaching, and online education are used interchangeably. Finch and Jacobs [19] define online education as any educational model where teachers and students are separated by time and geography. Larreamendy-Joerns and Leinhardt [20] consider online learning as education received via public, private, or internal networks. He [21] suggests that online teaching refers to a new educational model leveraging communication technology, promoting changes in the education system distinct from traditional face-to-face teaching. While providing a unified definition remains challenging, online education typically involves the separation of teaching and learning in time or geography, as well as the use of technology. In this study, online teaching refers to learning activities conducted without time and space constraints, utilizing network technology and platforms distinct from traditional classrooms. It encompasses various formats, including synchronous and asynchronous classes, allowing flexibility in time and space constraints.

Many studies have delved into exploring satisfaction with online teaching, often employing various theoretical models to investigate online satisfaction. For instance, some studies have utilized the customer satisfaction model [22]. Research on customer satisfaction has a long history. According to an exploration and review of existing studies, the “customer satisfaction model” was initially applied to the fields of goods consumption and services [23]. Later, the model was gradually introduced into the education industry. In 1994, the US company Noel-Levitz developed five different survey questionnaires for different universities to conduct a satisfaction survey of American university students (National Student Satisfaction and Priorities Report, (https://files.eric.ed.gov/fulltext/ED490031.pdf, accessed on 20 August 2021)). The survey had a wide range and large scale, and the results were recognized by the country, providing methods and tools for evaluating higher education quality correctly.

Lee and Mendlinger [24] used structural equation modeling (SEM) to analyze more than 800 students from the United States and South Korea, finding that perceived self-efficacy had a significantly positive correlation with online course acceptance and satisfaction. There were significant differences between learners in the two countries, but the scope of the differences could not be determined due to cultural and individual differences. Seiver and Troja [25] studied the relationship between belongingness, autonomy, control, and online learning satisfaction and success and found that belongingness was the most critical factor for improving satisfaction and promoting success. Kuo et al. [26] investigated a regression model for student satisfaction that incorporated student characteristics (including three types of interaction, internet self-efficacy, and self-regulated learning) along with class-level predictive factors (such as course category and academic course). The results showed that learner–content interaction was the most critical factor in improving student satisfaction, while the learner–learner interaction factor in online course settings may not be important. Subrahmanyam [27] used SEM with 1439 questionnaires from four public universities in India as effective samples, proving a significant correlation between service quality and learner satisfaction, continued use intention, and learning motivation, encouraging students to actively participate in the learning process to improve learning quality. Hong et al. [28] collected data from 433 learners and used SEM to verify that the six components of self-regulated online learning were negatively correlated with the perceived online learning inefficiency, with task strategy or emotion regulation showing a stronger correlation with learning inefficiency. Therefore, more attention should be paid to the influencing factors of online learning satisfaction.

A search using “online learning (e-learning, etc.)” and “satisfaction” as keywords in Web of Science reveals that most related studies are empirical analyses. Guo and Li [29] identified that the factors receiving the most attention in current research encompass “individual characteristics of students, technological tools, and interpersonal interactions”. Additionally, the variables most investigated are “self-efficacy, motivation, perceived ease of use, perceived usefulness, expectations, and social presence” [29]. Research on satisfaction in online learning has recently focused on several specific areas. For example, some researchers explored undergraduate students’ satisfaction with utilizing learning management systems in a blended learning setting [30]. Liu [31] investigated strategies to enhance satisfaction among Chinese college students in the context of online English teaching. Some researchers have explored the factors shaping the emotional experiences of Chinese undergraduate students in online English as a foreign language learning environments during the COVID-19 pandemic [32]. Their findings indicated that while perceived teacher internet and communication technology competence positively influenced enjoyment, it had a detrimental effect on anxiety and boredom. Some scholars have explored the correlation between engagement, readiness, and satisfaction in a synchronous online second language learning setting [15]. Their findings indicate that active engagement among L2 learners can significantly contribute to heightened satisfaction levels throughout a semester of synchronous remote learning [15]. Therefore, previous research on factors influencing learner satisfaction has mainly focused on exploring broad dimensions such as learner personal factors, teacher personal factors, learning resources, and platform services. However, recent studies have started to gradually refine and delve deeper into these dimensions, investigating deeper factors, such as a sense of belonging, autonomy, and emotional regulation, which impact learner satisfaction.

### 2.2. Theoretical Frameworks

Cardozo [23] proposed the concept of customer satisfaction and used a customer satisfaction model to analyze and measure it. The results showed that consumers’ feelings about product use were influenced by their expectations and payoffs. When consumers pay less, the smaller the gap between the perceived value of the product and their expectations; conversely, the higher they rate the product, the lower the rating. Lauterborn [33] proposed the 4C theory, which promoted the shift from the traditional marketing 4P theory (product, price, promotion, and place) to customer-centricity. The 4C theory emphasizes customer value, cost, communication, and convenience. Fornell et al. [34] constructed the American Customer Satisfaction Index (ACSI) model (Supplementary S1), which is the most salient model in marketing. Through continuous evolution and improvement, ACSI has become a standardized and scientific model and is currently the most widely used satisfaction model. To measure customer satisfaction, data are generally obtained by asking consumers questions or distributing questionnaires, and the obtained data are used to analyze the difference between user expectations and actual feelings about the product.

The ACSI model consists of six variables: customer expectations, perceived quality, and perceived value as the antecedents of satisfaction, and customer complaints and loyalty as the consequences of satisfaction. These variables are interrelated, and they collectively influence customer satisfaction [34].

**Figure 1:**
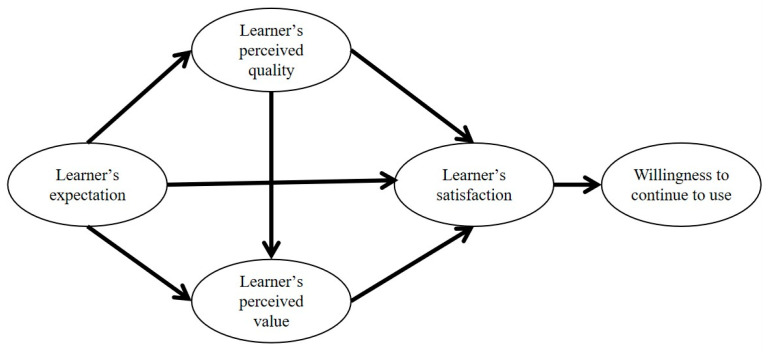
The Model of Chinese Learners’ Satisfaction in Online Courses.

It is widely acknowledged that learners can be viewed as consumers or experiencers of online courses, and their satisfaction with the course directly affects their willingness to continue using it. Given the peculiarities of educational research, this study adapts the ACSI model to explore the satisfaction of online Chinese language learners. The variables in the ACSI model were adjusted for this study. Specifically, “learner’s expectation” replaced “customer’s expectation”, “learner’s perceived quality” substituted “customer’s perceived quality”, “learner’s perceived value” replaced “customer’s perceived value”, “learner’s satisfaction” replaced “customer’s satisfaction”, and “learners’ willingness to continue to use” substituted “customer’s loyalty”. The variable of “customer complaints” was removed. This decision stemmed from the normalization of engaging in online courses for many learners during the COVID-19 period, where dissatisfaction may not necessarily lead to the discontinuation of using online courses, particularly among student populations and overseas learners who may lack alternative learning avenues. Hence, the variable of learners’ complaints was deemed redundant. Finally, a Model of Chinese Learners’ Satisfaction in Online Courses was constructed with learner expectations, perceived quality, and perceived value as antecedent variables, and learner’s willingness to continue to use as the consequence variable of learner satisfaction. Figure 1 displays the Model of Chinese Learners’ Satisfaction in Online Courses.

This study proposed the hypothesis that learner expectations have an impact on perceived quality, which occurs before learner perceived quality. Only after expectations are formed and perceived quality is obtained can learners determine whether the course has learning value. Perceived value is affected by expectations and perceived quality. Learner satisfaction can only be achieved after expectations, perceived quality, and perceived value are considered, and therefore, satisfaction is influenced by expectations, perceived quality, and perceived value. Satisfaction determines whether learners are willing to continue using or recommending the course.

(1) Research hypothesis based on individual differences of learners:

Learner characteristics may affect learner satisfaction [35]. Previous research has shown that individual characteristics can significantly impact learner satisfaction in online education settings. For example, studies have found differences in satisfaction levels between male and female learners [36], as well as between learners from different countries [37] or age groups [38]. Similarly, factors such as academic major, proficiency level in the target language, prior experience with online courses, and motivation to learn may affect how learners perceive the quality of online courses and their overall satisfaction with the learning experience. These factors may influence how learners perceive and engage with online Chinese language courses, ultimately affecting their satisfaction levels. Based on this, the study proposes the following hypotheses:

**H1.**  *Individual differences (gender, country, age, major, Chinese proficiency, prior experience with offline Chinese courses, platform usage, weekly learning hours, and learning motivation) may lead to variations in learner satisfaction.*

(2) Research hypothesis based on potential variables:

#### 2.2.1. Learner Expectations

Learner expectations refer to the learners’ anticipated feelings towards the course before taking the online course, including expectations for teaching content, knowledge acquisition, lecturers, and platform usage. Some researchers found that students’ expectations, perceived quality, and perceived value are key factors influencing satisfaction [39]. Among these, students’ expectations, derived from their past experiences and expectations of educational services, play a significant role in satisfaction. Additionally, service quality and perceived value also have a significant impact on satisfaction. Therefore, educational administration should prioritize and strive to enhance the quality of educational services to meet students’ expectations and improve satisfaction [39]. The fulfilment of expectations significantly explains satisfaction and is well accounted for by perceived quality dimensions, underscoring its mediating role between quality and satisfaction [40]. According to the research [39], student expectations have a direct and positive impact on perceived value. Based on the findings of the existing literature, this study proposes the following hypotheses:

**H2.**  *Learner expectations have a significant impact on learner perceived quality.*

**H3.**  *Learner expectations have a significant impact on learner perceived value.*

**H4.**  *Learner expectations have a significant impact on learner satisfaction.*

#### 2.2.2. Learner Perceived Quality

Learner perceived quality refers to the learners’ feelings towards the quality of the teacher’s qualifications, teaching content, independent learning, and platform services when they have studied or are studying an online course. Perceived quality transitions from a technical perspective, focusing on objective quality from the service provider’s standpoint to a more subjective approach based on customer perceptions, encompassing external or provision dimensions [39]. Scholars generally agree that students are the primary clients of educational activities [41]. Perceived quality is a key determinant of perceived value. When learners perceive the quality of online courses to be high, they are more likely to believe that the courses provide valuable benefits and outcomes. This study proposes the hypothesis that learners’ perception of online Chinese language course quality influences both their perceived value of the courses and their satisfaction levels. Given this context, this study proposes the following hypotheses:

**H5.**  *Learner perceived quality has a significant impact on learner perceived value.*

**H6.**  *Learner perceived quality has a significant impact on learner satisfaction.*

#### 2.2.3. Learner Perceived Value

Learner perceived value refers to the learners’ feelings towards whether online teaching can help improve learning effectiveness, learning interest, and learning motivation when they have studied or are studying an online course. Previous research revealed that core service quality and perceived value emerged as the primary determinants of customer satisfaction, whereas relational service quality played a significant albeit less prominent role [42]. Perceived value refers to the benefits or outcomes that customers obtain in relation to the total costs incurred during a purchase, which encompass both the price paid and associated expenses [39]. Essentially, it represents the disparity between perceived benefits and costs. The research found that students’ evaluations of interactivity, course content quality, and course design quality exerted significant and favorable effects on their perceived usefulness, confirmation, and satisfaction with the cloud-based e-learning platform [43]. These factors subsequently influenced their intention to continue using the system, either directly or indirectly. In the context of online education, learners’ satisfaction with courses is closely tied to their perception of the value provided by the courses. Considering this context, the study proposes the following hypothesis:

**H7.**  *Learner perceived value has a significant impact on learner satisfaction.*

#### 2.2.4. Learner Satisfaction

The concept of satisfaction was proposed by Cardozo [23], referring to the psychological state of pleasure that occurs when a person’s expectations are met. Martin [44] considers satisfaction to be the degree of correlation between a person’s feelings about what they have obtained and their expectations. The more closely the feeling matches or exceeds expectations, the higher the satisfaction. Satisfaction analysis was initially applied mostly in the business sector, but scholars gradually introduced the concept of satisfaction into other fields, such as medicine and education [45,46]. Martin and Bolliger [47] describe online learner satisfaction as the achievement of a student’s expectations and feelings of contentment towards various elements in the online learning setting, including the learner, instructor, course content, program structure, and organizational factors. In this study, learner satisfaction is defined as the psychological feeling that learners experience during or after the learning process, which increases as the degree of satisfaction with their learning needs increases. In addition, learner’s willingness to continue to use refers to the learners’ intention to continue choosing or recommending online courses after the course has ended. Research has consistently shown a positive relationship between satisfaction and continued usage intentions in online learning environments [14]. Given this context, this study puts forward the following hypothesis:

**H8.**  *Learner satisfaction has a significant impact on learner’s willingness to continue to use.*

## 3. Methodology

### 3.1. Participants and Data Collection

This study employed a combination of online and paper-based questionnaires for data collection. Online questionnaires were administered through the Wenjuanxing platform (https://www.wjx.cn/; accessed on 19 July 2021), while paper questionnaires were distributed to learners who found it inconvenient to use the online format. The survey targeted second language learners who have studied or are currently studying online Chinese courses without imposing restrictions based on nationality, gender, age, or learning platform, aiming to collect a broad range of feedback on the learning experiences and satisfaction levels of second language Chinese learners with online Chinese courses. Chinese courses are either compulsory or elective courses for the participants.

The respondents of the 203 valid questionnaires included international students from universities, high school students, and foreign residents. This study specifically focused on investigating online Chinese language courses for second language learners. These courses were offered as part of the curricula of the participants. Table 1 presents an overview of the personal information of the participants.

According to Table 1, there is a slight male predominance among the participants, accounting for 55.17%, compared to 44.83% for females. Regarding country of origin, learners come from 17 countries, mainly including Russia, Japan, and Thailand. This study categorizes these countries into two groups: developed and developing countries, with developed countries accounting for 46.31% and developing countries for 53.69%. The classification of developed and developing countries was based on acknowledged assessment metrics, such as GDP per capita, Human Development Index (HDI), and technological advancement.

In terms of age, all four age groups were involved, with only 16.26% of participants aged 30 and above, indicating that most participants were young adults between 18 and 30 years old. The age intervals were chosen based on common demographic categories used in educational research and to ensure a comprehensive representation of different age groups among the learners. For example, according to the United Nations Convention on the Rights of the Child, children are defined as individuals under the age of 18.

The participants’ majors are primarily categorized into two groups: Humanities and Social Sciences and Science and Engineering. The Humanities and Social Sciences category constitutes 41.38% of the cohort, encompassing fields such as social sciences, language and literature, philosophy, history, etc. Meanwhile, the Science and Engineering category makes up 58.62% of the cohort and includes disciplines like engineering, natural sciences, mathematics, computer science, etc. These major categories cover a wide range of academic disciplines, reflecting the diversity of participants’ academic backgrounds.

For Chinese proficiency, HSK (Hanyu Shuiping Kaoshi) exams are standardized tests administered by Hanban, a non-profit organization affiliated with the Chinese Ministry of Education, to assess non-native speakers’ proficiency in Mandarin Chinese. The classification into different HSK levels reflects learners’ proficiency levels, with higher levels indicating greater proficiency. Regarding Chinese proficiency, only 7.39% of participants have not passed the HSK exams, while HSK levels 5–6 account for 17.24%, HSK levels 1–2 and 3–4 account for 37.93% and 37.44%, respectively.

In terms of offline Chinese course experiences, more than 70% of participants have studied offline Chinese courses, which provides an opportunity to compare the effectiveness of online and offline courses. This aims to further understand students’ satisfaction with online teaching and their willingness to continue using online courses, helping to address the third research question.

Regarding platform usage, Tencent Meeting, DingTalk, and Xuexi Tong are the most frequently used platforms, accounting for 22.17%, 25.62%, and 24.63%, respectively. MOOC and Bilibili account for 16.75% and 8.37%, respectively. Five participants chose “Other platforms”, with ZOOM accounting for only 2.46%.

In terms of weekly study hours, 27.09% of participants study for more than five hours, while 37.44% study for 3–5 h, indicating that over 50% of the participants are experienced online course users and relatively familiar with the settings and operations of online courses.

Regarding learning motivation, the questionnaire provided nine options, including “improving Chinese language proficiency and communication skills” and “job-seeking needs”, and other options, which were categorized into internal and external motivations. Internal motivations accounted for 45.81%, while external motivations accounted for 54.19%.

Overall, the sample’s relative representativeness is supported by its diverse composition across various demographic factors and learning experiences, thereby enhancing the generalizability of the study findings.

### 3.2. Questionnaire Design

The data for this study were gathered through a questionnaire and structured interviews, chosen due to their prevalent use in research employing SEM. Questionnaires are widely regarded as the most reliable method for measuring the connections among constructs within the research model [14,48]. This research used an anonymous questionnaire to investigate participants’ satisfaction and their willingness to continue learning online courses. This method was designed to address the research questions (RQ1–RQ3) and hypotheses (H1–H8).

Based on the Model of Chinese Learners’ Satisfaction in Online Courses, the questionnaire items were designed and refined with reference to previous questionnaires [49,50]. The latent variables were proposed in the model. The questionnaire mainly consists of two parts: an investigation of the learner’s personal information (Part I) and an investigation of learner satisfaction (Part II). Part I includes the exploration of gender, nationality, age, major, Chinese proficiency, course platform, weekly duration of online courses, motivation for selecting the course, and prior experience with offline Chinese courses. Part II mainly consists of observation variables corresponding to five latent variables. It consisted of questions designed to measure the research model’s constructs, such as learner expectations, perceived quality, perceived value, learner satisfaction, and willingness to continue learning online courses. The specific variables and questionnaire items are shown in Supplementary S2. The questionnaire utilized a five-point Likert-type scale spanning from “strongly disagree (1)” to “strongly agree (5)”. The questionnaire consisted of 28 items to measure the five variables in the Model of Chinese Learners’ Satisfaction in Online Courses. In addition to the 28 scale questions, the questionnaire also includes one non-scale single-choice question to collect more detailed data for analysis.

Frequency and percentage were calculated to analyze the demographic information of Part I of the questionnaire. In Part 2 of the questionnaire, numerical scores based on 5 Likert scale items were analyzed using SPSS 26.0 (Statistical Package for Social Sciences). Descriptive statistics (mean scores, standard deviation, *t*-test, and one-way Analysis of Variance (ANOVA), LSD, *p* value) were used to indicate the learners’ individual differences toward learning online Chinese courses in the five dimensions. For example, gender difference analysis was verified using an independent sample *t*-test. If the *p* values of gender in the five dimensions of learner expectation, perceived quality, perceived value, learner satisfaction, and willingness to continue using are greater than 0.05, this indicates that there is no significant difference between gender in these five dimensions. This shows that gender factors will not affect the satisfaction of online teaching Chinese as a foreign language. The personal differences of learners were statistically analyzed, and SPSS was used to test the reliability and validity, difference analysis, correlation analysis, and regression analysis of the data. Goodness-of-fit test results for the regression of each factor were analyzed. The content analysis technique was employed to analyze the open-ended questions in the questionnaire.

The questionnaires and interviews were both conducted in Chinese, as the target participants were learners of Chinese as a second language. Due to the fact that the respondents of the questionnaire are second language learners, English translations are provided for the Chinese items to facilitate comprehension (Supplementary S3). To ensure the comprehensibility of the questionnaire or interview, several pre-tests were conducted by 20 participants prior to the main data collection phase. The purpose of these pre-tests was to identify any ambiguities or difficulties in understanding the question prompts. Based on the feedback from the pre-tests, necessary revisions and adjustments were made to the questionnaire and interview outline to enhance clarity and comprehensibility.

In the formal experiment, a total of 225 questionnaires were collected, among which 203 were valid questionnaires. The effective response rate of the questionnaire was 90.2%. This study used these 203 questionnaires as the analysis data. The main advantage of using SEM lies in exploring the causal relationships between latent variables. The researchers first constructed a model and then collectively analyzed the findings, assessing the proposed seven hypotheses (H2–H8), and providing the path coefficients. The proposed model was evaluated, and the structural coefficients were estimated using SPSS. This proposed model can be viewed as an integration of multiple regression analyses. It facilitates analysis of both direct and indirect relationships among multiple variables.

### 3.3. Interview Design

To deepen understanding of second language learners’ perspectives on instructors, teaching content, and online platforms within the course, interviews were conducted in this study to explore additional aspects not covered in the questionnaire. The semi-structured interview data were transcribed and analyzed using content analysis methods. Coding analysis was then applied to extract significant concepts from the language used and interpret them within the main themes [51]. This approach addressed Research Questions 1–3. In-depth interviews with learners highlighted the understanding of how the main factors influence students’ satisfaction and their willingness to continue using online Chinese courses.

Five learners who volunteered after completing the questionnaire survey were interviewed. This enabled a comparative analysis to better understand their satisfaction with online teaching and their willingness to continue online courses.

The interviewers were trained to ensure consistency and standardization when conducting the interview. Those interview participants were selected based on their diverse backgrounds and experiences with Chinese language learning. They come from various countries, representing a mix of cultural and linguistic backgrounds. Additionally, their Chinese proficiency levels ranged from HSK 3 and above, ensuring that they possessed sufficient language skills to effectively express their experiences and opinions. The details of the interviewees, including their demographics and language proficiency, are provided in Supplementary S4.

The interview mainly focused on the following questions:

1. Which type of teaching mode do you prefer, online or traditional classroom teaching? Why?
2. What was the biggest problem you encountered in online courses, and have you encountered it in traditional classroom courses?
3. What suggestions do you have for online courses (including but not limited to teachers, platforms, course content, etc.)?

## 4. Results

### 4.1. Reliability and Validity Test

Examining a questionnaire’s reliability ensures whether the data obtained are accurate and can truly reflect the actual situation. SPSS 26.0 was used to conduct a reliability test of the questionnaire. The detailed results are shown in Supplementary S5. The reliability coefficients of the five variables are all greater than 0.8, and the overall reliability of the questionnaire reaches 0.947, indicating that the questionnaire has a high reliability.

The Kaiser–Meyer–Olkin (KMO) test and Bartlett’s sphericity test were used to determine the validity. Factor analysis results showed that the KMO value was 0.938. Significance was found in Bartlett’s sphericity test (*p* = 0.000). This indicates a strong correlation between the variables, which is very suitable for factor analysis. The detailed results are shown in Supplementary S6.

### 4.2. Statistical Analysis of Differences

To investigate whether individual differences impact learners’ expectations, perceived quality, perceived value, satisfaction, and intention to continue learning online courses, this section presents the statistical results of gender, countries, age, academic discipline, Chinese proficiency level, experience with offline Chinese classes, platforms, study duration, and motivation across the five dimensions. The summarized statistical results are shown in Table 2, with specific information for each factor listed in separate tables, as shown in Supplementary S7.

Table 2 shows that there is no significant difference in terms of learner satisfaction in online Chinese language teaching based on gender (*p* > 0.05). No significant differences were found among learners from developed or developing countries in terms of learner expectations, perceived quality, perceived value, satisfaction, and willingness to continue using online courses (*p* > 0.05). There were no significant differences in learner expectations, perceived quality, and perceived value among different age groups (*p* > 0.05), while significant differences were observed in learner satisfaction (*p* = 0.02) and willingness to continue using (*p* = 0.047) among different age groups. Younger learners (<18 years old) showed lower satisfaction and willingness to continue using compared to other age groups. Regarding discipline, no significant differences were found in learner satisfaction between Humanities and Social Sciences and Science and Engineering groups (*p* > 0.05). No significant differences were found in learner expectations, perceived quality, perceived value, and willingness to continue using based on Chinese language proficiency levels (*p* > 0.05). There was a significant difference in satisfaction levels (*p* = 0.010), with higher satisfaction as Chinese proficiency level increased. Significant differences were found in learner expectations, perceived quality, perceived value, satisfaction, and willingness to continue using online courses between learners with and without previous offline learning experience (*p* < 0.05). Learners with prior offline experience had lower satisfaction scores. There was a significant difference in perceived quality among different online learning platforms (*p* = 0.018). Specifically, StudyTube users had significantly lower perceived quality scores than users of other platforms. No significant differences in perceived value were found among learners with different weekly study times (*p* = 0.056). There were significant differences in expectations, perceived quality, satisfaction, and willingness to continue using the course among learners with different weekly study times (*p* < 0.05). Learners studying for five or more hours per week showed lower scores in these dimensions. No significant difference was found in learner expectations, perceived quality, perceived value, satisfaction, and willingness to continue using among participants with different learning motivations (both internal and external motivations).

These findings provide insights into the factors influencing learner satisfaction and engagement in online Chinese language courses. The above results showed that the proposed hypotheses were supported, and the research questions were answered comprehensively.

### 4.3. Correlation Statistical Analysis

This section presents the results of a correlation analysis, which was carried out with the aim of analyzing the degree of linear correlation between two variables. Pearson’s coefficient was used as the test index (Table 3).

As shown in Table 3, all five variables, namely learner expectations, perceived quality, perceived value, satisfaction, and intention to continue using the online course, are positively correlated with each other (correlation coefficient > 0), and the strongest correlation is observed between satisfaction and perceived quality (coefficient value = 0.570).

### 4.4. Regression Analysis

To determine the causal relationships between variables, regression analysis was conducted. This study used both univariate and multivariate linear regression analyses to describe the causal relationships between variables. The results of the goodness-of-fit test are shown in Table 4.

Specifically, to explore the impact of learners’ expectations on perceived quality, this study incorporated learners’ expectations as independent variables and perceived quality as the dependent variable, conducting a univariate regression analysis. The goodness-of-fit test results for the regression of perceived quality in Table 4 show that the regression model of perceived quality is significant (*F* = 63.916, *p* = 0.000). This indicates that further analysis can be conducted. The correlation coefficient *R* represents the strength of the relationship between the independent and dependent variables. *R*^2^ multiplied by 100% represents the percentage of variance in the dependent variable explained by all independent variables as a whole. Adjusted *R*^2^ provides a more accurate estimate of the dependent variable, and a value closer to 1 indicates a better fit of the model. In Table 4, *R* = 0.491, *R*^2^ = 0.241, and adjusted *R*^2^ = 0.237, indicating that 23.7% of the variation in perceived quality can be explained by changes in learners’ expectations, and the model has an acceptable fit. The Durbin-Watson value of 2.078 suggests that the model is well constructed.

To explore whether learners’ expectations and perceived quality influence perceived value, this study set learners’ expectations and perceived quality as independent variables and perceived value as dependent variables, conducting stepwise regression analysis. The goodness-of-fit test results for the regression of perceived value in Table 4 show that both regression models are significant (*p* = 0.000). As the *R*^2^ and adjusted *R*^2^ values gradually increase from Model 1 to Model 2 when perceived value was the dependent variable, the regression equation of Model 2 is better. The Durbin–Watson value of 2.112 in Model 2 indicates good independence among variables. This indicates a well-constructed model. The *R* = 0.572, *R*^2^ = 0.327, and adjusted *R*^2^ = 0.320 indicate that 32.0% of the variance in perceived value can be explained by changes in learner expectations and perceived quality.

To investigate whether learners’ expectations, perceived quality, and perceived value influence learner satisfaction, this study included learners’ expectations, perceived quality, and perceived value as independent variables and satisfaction as the dependent variable in a stepwise regression analysis. The goodness-of-fit test results for the regression of satisfaction show that all three regression models are significant (*p* = 0.000). The *R*, *R*^2^, and adjusted *R*^2^ values increased progressively from Model 1 to Model 3 when satisfaction was the dependent variable. This indicates that Model 3 exhibits the best regression equation, with *R* = 0.672, *R*^2^ = 0.451, and adjusted *R*^2^ = 0.443. This indicates that the model fits well, and 44.3% of the variation in learner satisfaction can be explained by changes in perceived quality, perceived value, and learner expectations. The Durbin–Watson value is 1.908, which is close to 2, indicating that the model is well constructed.

To explore the influence of satisfaction on learners’ willingness to continue using online course, this study included learner satisfaction as an independent variable and the willingness to continue using as the dependent variable in a univariate regression analysis. The goodness-of-fit test results for the regression of willingness to continue using shows that the regression model was significant (*F* = 78.395, *p* < 0.05). *R* = 0.530, *R*^2^ = 0.281, and adjusted *R*^2^ = 0.277, indicating that 27.7% of the variation in learners’ willingness to continue using could be explained by changes in learner satisfaction.

Table 5 summarizes the regression coefficients (Beta) and significance test results for each independent variable on the dependent variable. If the VIF value is less than 5, this indicates that there is no multicollinearity problem in the model, and the model is well constructed.

As shown in Table 5, all of the VIF values are less than 5, indicating no multicollinearity issue in the models. The standardized beta coefficient for learners’ expectations of perceived quality is 0.491 (*t* = 7.995, *p* = 0.000), indicating that learners’ expectations have a significant positive effect on perceived quality.

When perceived value was set as the dependent variable, both learner expectations and perceived quality had significant effects on perceived value, with regression coefficients of 0.397 and 0.260, respectively (*p* < 0.05).

The regression coefficients for satisfaction show that when learner satisfaction was the dependent variable, the VIF values of model 3 range from 1.418 to 1.552. The values are below 5, indicating no multicollinearity issues among the variables and indicating a well-constructed model. The regression coefficients for perceived quality, perceived value, and learner expectations on learner satisfaction are 0.341, 0.250, and 0.235, respectively. Their significance levels are all below 0.05 (*p* = 0.000), indicating significant positive effects of learner expectations, perceived quality, and perceived value on learner satisfaction.

The regression coefficient for the willingness to continue using online course is shown in the last column of Table 5. The coefficient of learner satisfaction was 0.530 (*p* = 0.000), indicating a significant positive effect of learner satisfaction on willingness to continue using online courses.

**Figure 2:**
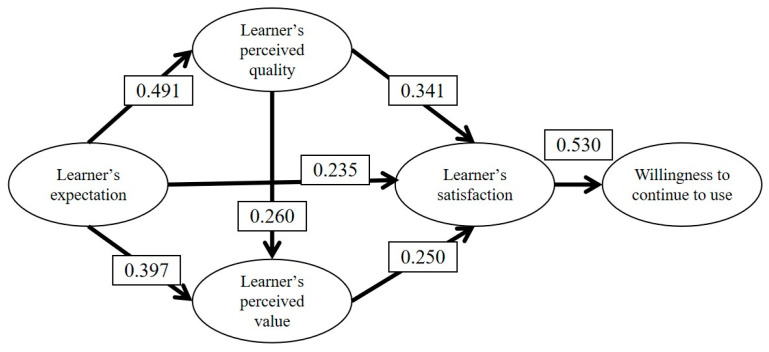
Path analysis of Model of Chinese Learners’ Satisfaction in Online Courses.

Combining the results of linear regression analysis, the validated Model of Chinese Learners’ Satisfaction in Online Course was created (Figure 2).

As shown in Figure 2, the proposed structural model was utilized to verify the hypotheses by assessing the path coefficients. The numbers, such as 0.491, depicted in the schema, were derived from the regression coefficients corresponding to each factor. These numbers represent standardized coefficients (beta). In conclusion, the proposed Model of Chinese Learners’ Satisfaction in Online Courses and the hypothesized relationships among variables were supported by the above results.

### 4.5. Statistical Analysis of Non-Scale Items

At the end of the questionnaire, participants were asked to respond to a single non-scale question: “Which mode of learning Chinese do you prefer?” The options were “offline mode”, “online mode”, “offline and online mixed mode”, and “no mode matters” The results for each option are presented in Table 6.

Table 6 shows that most participants preferred offline learning mode (42.86%), followed by mixed mode (33.50%). This indicates that although online learning has become a trend, most learners still prefer the traditional offline teaching mode. Currently, traditional teaching has not been replaced by online teaching and still holds an important position. To explore the advantages and disadvantages of online teaching compared to traditional teaching, as well as areas that require improvement, this study conducted in-depth interviews with some Chinese language learners.

### 4.6. Interview Survey Results

This section presents interview results from five participants regarding their preferences and experiences with online and traditional classroom learning. The interviews were analyzed to explore the individual differences impact learner satisfaction with online Chinese courses and identify factors that influenced learner satisfaction with online courses. The interviews of five participants were transcribed and analyzed. The responses to the questions are as follows:

For Question 1:

A: “I prefer attending classes at school because I feel that online classes reduce opportunities for communication with teachers and classmates, resulting in less time to practice speaking. However, online courses are more convenient and save commuting time. Sometimes, I can also sleep in”.

B: “I prefer offline learning because there are often many interesting games in Chinese classes, which are less present in online learning activities. Moreover, poor network connections can cause interruptions that make it difficult to understand the lessons. Staring at the screen for extended periods also causes eye fatigue and distraction”.

C: “I prefer learning in a classroom. Since I returned to China, I have been taking online courses at home, and I really miss the experience of attending classes on campus. Sometimes, poor internet connectivity and the international time difference during classes makes me very tired. Online classes also require greater self-discipline to understand the material”.

D: “I prefer to learn in the classroom. Switching to a new platform for online classes can cause difficulties for both teachers and students and can be disruptive to learning”.

E: “I prefer offline classes. Staring at the screen for extended periods causes eye fatigue, and sometimes my eyes feel sore. Sitting in front of the computer for long periods also causes discomfort in my back”.

For Question 2:

A: “Efficiency is low in online courses because of the replay function. When I am bored, I tend to get distracted. Also, teachers cannot notice my state. Sometimes, relying too much on the replay function leads to a lack of focus and low learning efficiency. This is not a problem in offline classrooms”.

B: “There is too little communication with the teacher in online courses. In offline courses, teachers communicate more with the class. In online courses, the teacher asks fewer questions to students and assigns more home C: “Sometimes, some students can’t understand the lesson, and the teacher won’t repeat the lesson. In offline classes, the teacher’s voice is clearer, and students are more willing to ask questions. The time difference also affects my mental state. This is not a problem in offline classes”.

D: “In online courses, the teacher asks fewer questions to students and assigns more homework with less feedback”.

E: “The network signal is unstable, and sometimes I can’t hear the teacher clearly, and there is no follow-up question”.

For Question 3:

A: “Some courses do not have replay functions. I hope that the replay function can be added because sometimes poor network connectivity can result in missing part of the class”.

B: “Compared to offline courses, there is less communication with teachers in online courses. I hope teachers can ask more questions and encourage more interaction between classmates”.

C: “I hope that the platform’s functions can be more user-friendly. I also hope that the course schedule can be arranged reasonably to minimize the impact of the time difference on learning status. However, compared to these D: Teachers can give students more time for oral communication in class, allowing them to practice speaking “more””.

E: “I hope the teacher can use the network platform that is more simple and not too laggy”.

Based on interviews with five participants, it was found that they generally preferred offline courses over online courses. While online courses offer advantages such as convenience and efficiency, there are also several areas of concern. Specifically, the following issues were identified with online courses: First, there is a decrease in opportunities for verbal communication with teachers and peers, which hinders oral practice and discussions. Second, there are fewer games and activities for interaction in class, while assignments have increased, leading to a decrease in classroom engagement. Third, technical problems such as network latency and time differences can negatively affect the quality of the learning experience and students’ emotions. Fourth, extended periods of online learning can cause discomfort in the eyes and other parts of the body. Fifth, students may experience lower efficiency and motivation. Sixth, teachers and peers may be unfamiliar with the platform operation, and the replay function may require improvement.

## 5. Discussion

The results are consistent with the experimental hypothesis predicted by the Model of Chinese Learners’ Satisfaction in Online Courses. Learner satisfaction is significantly influenced by individual differences, including age, Chinese proficiency level, duration of learning, and prior experience with offline Chinese courses. No significant difference in learner satisfaction in online Chinese language teaching was found based on gender. These results are, to some extent, corroborated by some previous studies. Al-Azawei and Lundqvist [52] investigated the learner differences in perceived satisfaction with online learning. They discovered that the perception of usefulness was the most accurate predictor, whereas online self-efficacy and ease of use did not demonstrate a direct impact on satisfaction. Additionally, neither learning styles nor gender diversity had a direct impact on the dependent factors. Lu and Chiou [53] found that students’ perceptions of predictors and their satisfaction with the e-learning system were significantly influenced by both gender and job status. Several studies have indicated variations in online learning satisfaction based on gender. For example, some studies have observed higher satisfaction among female students compared to male students in e-learning subjects [36]. However, contrasting findings from another study revealed no statistically significant difference between the gender groups [54]. Furthermore, the relationship between predictors and online learning satisfaction is moderated by two contingent variables: student job status and learning styles.

The findings indicated that learner expectations exert a notable positive influence on perceived quality, which in turn positively affects perceived value. Moreover, learner satisfaction significantly boosts the likelihood of learners’ willingness to continue using online courses. The satisfaction of learners has a significant positive impact on their willingness to continue using online courses. These findings are consistent with the previous study [39]. The results revealed that the majority of learners still prefer traditional, offline courses, thereby indicating that online teaching still possesses shortcomings and deficiencies. These findings were consistent with the previous studies [55,56]. Based on the empirical analysis results obtained from the survey and interview in Section 4, several suggestions are proposed to improve learner satisfaction.

### 5.1. Emphasizing the Improvement of Online Chinese Language Learning Satisfaction among International Students in Universities

First, it is suggested that more attention should be paid to enhancing the satisfaction of online Chinese language learning among international students in universities. A previous study [37] found that student satisfaction is associated with factors such as residence location, prior experience with online learning, and the influence of friends or family using online learning resources. Satisfaction surveys conducted on learners of different age groups revealed that those aged below 18 and between 18 and 25 had relatively lower levels of satisfaction. On average, learner satisfaction increases with age, which may be related to the learning experience and goals of international students. There were some possible reasons. Firstly, it could be related to the learners’ educational background. Students in different age groups have varied educational experiences, with most learners under 18 and between 18 and 25 being students in school, for whom online Chinese courses are either compulsory or elective courses. On the other hand, learners over 25 are mostly working professionals for whom online Chinese courses serve as supplementary courses in their spare time. Compared to extracurricular tutoring courses, school courses are more difficult, require credits, and are more important. Younger learners experience more pressure from online learning, which may lead to lower satisfaction and, therefore, lower intention to continue using these courses compared to older learners.

Secondly, it may be related to learners’ achievement goals. Achievement goals can be divided into mastery goals and performance goals [57], with mastery goals focusing on knowledge acquisition and performance goals focusing on self-evaluation. Middle and high school students are more influenced by mastery goals and place greater emphasis on mastering knowledge, attaching importance to teacher–student interactions, and hoping for stricter supervision from teachers, thus resulting in lower satisfaction with online courses than older learners. On the other hand, online courses offer greater flexibility in learning and allow for the development of a personalized, flexible learning plan, making them more suitable for older working professionals, resulting in higher satisfaction with online courses among this group.

Most learners below 18 and between 18 and 25 are students in universities where Chinese language courses are mandatory or elective subjects. Compared with extracurricular Chinese tutoring classes attended by working adults, university students face greater difficulty in learning Chinese, as they have academic credits to earn and higher pressure to perform well in their studies. Therefore, they may not find the online learning process as easy and enjoyable, resulting in lower satisfaction.

Furthermore, the learning goals of university students lean towards knowledge acquisition, as revealed by the interviews. The interviewed students, who were all university students, expressed a preference for traditional teaching methods with supervision, more interaction with teachers and classmates, and assistance from teachers to improve their learning efficiency. According to the theory of interaction in online education, good interaction can help students effectively grasp knowledge. In summary, while online Chinese language courses for learners of all ages and from various channels require attention, international students in universities remain the primary group of learners of Chinese as a foreign language and deserve special attention to enhance their satisfaction.

### 5.2. Reasonable Course Arrangement Is an Effective Method to Improve Learner Satisfaction

#### 5.2.1. Reasonably Arrange Teaching Based on Different Chinese Proficiency Levels

Significant differences in learner satisfaction were observed among students with different levels of Chinese language proficiency. The mean satisfaction score for learners who have not passed the HSK exam was 3.39, whereas those who have passed HSK1–2, HSK3–4, and HSK5–6 had mean satisfaction scores of 3.77, 3.93, and 4.17, respectively. This suggests that learner satisfaction increases as their Chinese language proficiency level increases. Lower-proficiency-level learners may have lower satisfaction due to their limited language skills. This may hinder their ability to keep up with the pace of the class, resulting in lower learning efficiency and poor classroom performance. To address this issue, learners can be grouped based on their language proficiency levels. Different teaching plans, strategies, and methods can be adopted for different proficiency levels to promote teaching progress gradually. Additionally, teachers must focus on every student, especially those with lower proficiency levels, to ensure that they have the opportunity to speak up, practice their language skills, and solve common problems in the classroom.

#### 5.2.2. The Course Duration and Schedule Should Be Appropriate

Learners with different weekly study hours have significant differences in terms of their expectations, perceived quality, satisfaction, and willingness to continue using online courses. The least significant difference (LSD) post hoc test revealed that learners who study for more than 5 h per week have significantly lower scores in these four dimensions compared to other groups. Learners who study for 3–5 h per week have significantly lower satisfaction scores than those who study for less than one hour. This may be because learners who spend more time studying Chinese may find it challenging to cope with the daily pressure of long hours of studying, leading to negative attitudes towards online learning.

According to the interviewees, prolonged screen time can cause eye and back pain, leading to decreased satisfaction levels. Therefore, it is crucial to adjust the duration of online courses according to students’ needs, including weekly study hours and class time. Weekly study hours should be adjusted based on the students’ actual situation, learning objectives, and teaching syllabus. While class duration should be appropriate, providing enough time for students to take breaks and relieve eye, body, and brain pressure is necessary so as to enhance the satisfaction of online courses.

### 5.3. Improving Course Expectations and Reducing Upfront Costs Can Help Enhance Learners’ Perceived Quality and Value

This study found that the expectations of learners have a substantial and positive influence on perceived quality, while perceived quality, in turn, exerts a notable and positive impact on perceived value. This finding was consistent with the previous study. For example, Marimon et al. [40] found that fulfilment of expectations plays a mediation role between quality and satisfaction.

As with the results of the regression analysis in Section 4, the regression coefficient of learner expectation towards perceived quality is 0.491, indicating a positive effect. Learner expectations and perceived quality have a significant impact on perceived value with regression coefficients of 0.397 and 0.260, respectively. Thus, improving learner expectations plays a critical role in enhancing learner perceived quality and value. According to the customer satisfaction theory, the “4C” theory and the expectation gap theory, customer perceptions of products are influenced by expectations and costs. Therefore, course developers should focus on promoting course quality during pre-course promotion to ensure that learners’ course experience exceeds their expectations, leading to greater satisfaction and stronger motivation for continued learning. Additionally, course resources should be easily accessible, and the platform interface should be simple and convenient to lower the material, energy, and time costs for learners to acquire knowledge. This would enhance the perceived quality and value of the course and, in turn, improve course satisfaction.

### 5.4. Improving Course Quality Is the Key to Improving Learner Satisfaction

The correlation analysis reveals that learner expectations, perceived quality, perceived value, satisfaction, and willingness to continue using the platform are positively correlated with one another. Online learning satisfaction is most strongly correlated with perceived quality (Pearson’s coefficient is 0.570). The regression analysis shows that the regression coefficient values for perceived quality, perceived value, and learner expectations toward learner satisfaction are 0.341, 0.250, and 0.235, respectively. This indicates that perceived quality is the most critical factor affecting learner satisfaction. Thus, improving online course quality is the key to enhancing learner satisfaction. This is consistent with the findings of previous research [39].

#### 5.4.1. Improving the Quality of Teachers and Teaching Quality

Teachers play a dominant role in online classrooms. Improving their teaching quality and qualifications is a crucial pathway to enhancing student satisfaction. According to the interviewees, online courses have relatively fewer fun activities and games, reducing classroom interest. Furthermore, teachers may lack experience in online teaching and encounter new problems compared to traditional classroom teaching. For instance, teachers may rush through lessons to meet curriculum requirements, resulting in insufficient communication with students both inside and outside the classroom, as well as a lack of supervision over their completion of assignments. Additionally, teachers may lack familiarity with platform operations, leading to delays in course progress and impacting teaching effectiveness. Thus, Chinese language teachers should improve their teaching skills and qualifications to enhance teaching quality.

Firstly, it is crucial for teachers to possess advanced computer skills and to familiarize themselves with the functions of the online teaching platform beforehand. This will prevent any disruptions to the class caused by their lack of proficiency in operating the technology. Teachers should arrange more online activities that are both engaging and suitable for online teaching. They should try to keep their cameras on to observe the facial expressions and states of every student, which will allow for better supervision and communication. In addition to language teaching, the teaching of foreign languages requires the use of body language, facial expressions, and gestures as supplementary teaching methods. Teachers should provide students with more video and audio-based teaching materials to fully engage their senses and enhance their language learning outcomes.

Secondly, teachers should provide students with ample opportunities for interaction and communication with both their peers and the teacher. According to the theory of interactive online education, an appropriate interactive environment can facilitate effective knowledge construction, whereas a lack of interaction can impede it. Although research in information technology is currently popular, the success of online education ultimately depends on “education”. Teachers must focus on the real needs of their students and utilize information technology and interaction methods that are tailored to their situation to maximize the effectiveness of their teaching. Therefore, teachers must be responsive to and attentive to the needs of their students. They should actively engage in interactive activities, enhance teacher–student interaction, provide channels for student–student communication, and offer various high-quality learning resources to promote student–content interaction.

Teachers should avoid simply reading out the course materials and presentations and instead allow students to take center stage and actively participate in the class, fostering a lively classroom atmosphere and stimulating students’ interest in learning. Speaking is the most effective way to learn a language, so it is essential to increase opportunities for teacher–student interaction and encourage peer-to-peer communication. Teachers should also address students’ post-class questions and provide timely feedback.

Fourthly, in terms of homework, teachers should consider students’ actual situations and ensure that the homework is consistent with the class content. The amount of homework assigned should be appropriate, avoiding mechanical copying and reducing the workload on students.

Fifthly, for difficult courses, teachers should provide recorded lectures so that students can review the course content repeatedly to reinforce their learning outcomes. The learning resources used in class, such as videos, audio, and presentations, should be promptly sent to students for self-study purposes.

#### 5.4.2. Improving the Quality of Learner Autonomous Learning

According to the theory of autonomous learning, when learners are intrinsically motivated, they can independently develop and reasonably arrange learning plans. They are highly sensitive to the learning environment and possess improvisation skills. As a result, their learning becomes highly self-directed, leading to greater learning outcomes. While online courses provide greater flexibility to learners as they are not bound by time or location and do not incur commuting expenses, they do pose some challenges. Based on the interview, learners reported lower learning efficiency and reduced self-awareness due to a lack of teacher supervision while studying online. Moreover, they lacked opportunities to practice speaking Chinese, leading to poor learning outcomes. Therefore, it is urgent to address these issues and enhance learners’ awareness and quality of autonomous learning.

Firstly, learners should adopt a positive attitude towards online learning and take responsibility for their own learning. Since online courses are significantly different from traditional face-to-face classes, the role of teachers in monitoring learners’ progress is limited. Therefore, learners must improve their self-management skills, define their learning goals, and cultivate a positive attitude towards online learning while overcoming any negative emotions such as fear and anxiety.

Secondly, learners should improve their information technology literacy. Online learning has become a necessary learning method for many learners, and mastering the functionality and interface of the platform is an essential skill. While learning how to operate various intelligent learning tools, learners should also enhance their skills in acquiring and processing various types of information. They should be open-minded and accepting of online learning and courses from various channels to reap the best learning outcomes.

Thirdly, learners should strengthen self-monitoring and intervention and place greater emphasis on pre- and post-class work. Learners who preview the learning content in advance can identify the crucial and challenging points of the course, which can help them focus their learning in class. After class, they should consolidate their knowledge by reviewing and completing assignments promptly, using videos and other materials provided by the teacher or the platform to practice their pronunciation and improve their understanding of language concepts. Learners should not be afraid to communicate with their teachers and classmates in and out of class and should promptly provide feedback to their teachers and platforms if they have any questions.

#### 5.4.3. Enhancing the Quality of Platform Services

As an emerging teaching model that relies on network and information technology, the importance of platform service quality in online teaching is self-evident. The results showed there are significant differences in perceived quality among different platforms (*p* < 0.05). During the interviews, participants mentioned that sometimes network and platform lags can cause the course progress to be less smooth, resulting in poor learning outcomes and negative emotions among learners. Therefore, the platform for online courses also needs to strengthen its own service quality.

Firstly, it is crucial to reduce the lag of the network and online platform to ensure smooth classroom progress. Currently, there are numerous online course platforms, but many of them are still relatively new and may have some incomplete functions. The first issue that needs to be addressed is the problem of lag in the network and platform, which includes not only slow internet speed but also voice and image lag or failure, as well as insufficiently clear voice and image quality. This requires the platform to actively listen and respond to user feedback, as well as the continuous efforts of backend professionals to ensure the stability of platform operation.

Secondly, it is necessary to improve and optimize platform functions and design more user-friendly service platforms based on users’ needs. Some platforms have complicated operations and insufficiently concise and clear functions. Therefore, app developers need to improve and optimize platform functions based on the needs of platform users. Operation and course interfaces should minimize complex and less effective designs and add or optimize functions that learners need, such as course playback or recording functions, online assignment submission functions, pre-class and review reminder functions, etc. Meanwhile, platform bugs should be repaired promptly to avoid interfering factors that may affect learners’ online learning.

## 6. Conclusions

### 6.1. Findings

This paper contributes to the literature by constructing a model of Chinese learners’ satisfaction with online courses, drawing from empirical data collected from second language Chinese learners in Shanghai. The results elucidate the significant impact of factors such as learner expectations, perceived quality, and perceived value on online learning satisfaction and willingness to continue using online courses, thus offering empirical support for the theoretical advancement of satisfaction in second language learners’ online education. The findings hold practical significance for individuals involved in educational development, policy formulation, and implementation, with the goal of improving the quality of online courses by employing efficient strategies.

The empirical analysis revealed the following findings: (1) Individual differences among learners were found to be significant factors affecting satisfaction and willingness to continue using the online courses. These differences included age, Chinese proficiency level, prior offline Chinese learning experience, platform used, and weekly study duration. (2) In terms of latent variables, learner expectations had a significant positive impact on perceived quality, and both expectations and perceived quality had a positive impact on perceived value and satisfaction. (3) Satisfaction, in turn, had a significant positive impact on willingness to continue using the online courses. (4) In terms of a comparison between online and offline courses, the results of the interviews show that learners still preferred traditional offline teaching. Although online courses are more convenient and offer greater accessibility, there are some disadvantages to online courses. For example, these include reduced communication with teachers and peers, fewer interactive games and activities, increased homework workload, and teachers’ lack of proficiency in using the platform. Learners also reported discomfort due to prolonged screen time and lower motivation.

Based on these findings, this study proposes strategies for improving learner satisfaction, including focusing on the satisfaction of international students, properly scheduling online courses, enhancing learners’ expectations, reducing upfront learning costs, improving the quality of teaching, promoting self-directed learning, and improving the quality of network platform services. Specifically, there are more suggestions to enhance the satisfaction of Chinese language learners in online learning: First, tailor online courses to accommodate individual differences such as gender, country of origin, age, major, Chinese proficiency level, prior experience with offline Chinese courses, platform usage preferences, weekly learning hours, and learning motivation. Offer personalized learning pathways, resources, and support to meet diverse learner needs and preferences. Second, educate learners about realistic expectations for online courses to align them with the actual learning experience. Provide clear and transparent course descriptions, objectives, and outcomes to manage learner expectations effectively. Third, invest in improving the quality of online course content, delivery, and instructional design to meet learners’ expectations and enhance perceived quality. Fourth, emphasize the value of online courses by highlighting their relevance, applicability, and benefits to learners’ personal and professional development. Fifth, place a strong emphasis on learner satisfaction as a key indicator of course effectiveness and quality. Continuously gather feedback from learners through surveys, evaluations, and assessments to identify areas for improvement and address any issues or concerns promptly. Sixth, foster a supportive and engaging learning environment that encourages learners to continue using online courses. By implementing these recommendations, educational institutions and online course providers can enhance the satisfaction and willingness to continue learning online courses, ultimately improving the effectiveness and quality of online language education.

On a practical level, the research offers targeted improvement strategies for teachers and online course platforms, which hold crucial significance in enhancing the teaching quality and online learning experience for second language learners. The findings provide an important reference for improving the learning experience and teaching quality of second language learners in online courses, providing valuable insights for educational practices and online course design.

### 6.2. Limitations and Further Research

Research on learner satisfaction in online courses for teaching Chinese as a foreign language is a relatively new and emerging field. There are still some limitations that need to be addressed in future research. Firstly, the sample may not be representative, as the study only surveyed learners who have studied or are currently studying online Chinese courses in Shanghai, with limited coverage of learners from other regions. To enhance the representativeness of the sample, future research could expand the coverage of the survey to include more regions and learners. Secondly, the number of interviewees is relatively small, and the interview content is relatively simple. Future research could increase the number of interviewees and design more comprehensive and in-depth interview questions to gain a more holistic understanding of learners’ self-perceptions of online Chinese language courses. Thirdly, the study does not provide a detailed analysis of learners’ motivations, only categorizing them into internal and external motivations. In future research on learning motivation, specific analyses of each motivation can be conducted to provide more targeted satisfaction enhancement strategies for different learners. For instance, for learners who study Chinese due to an interest in Chinese language, culture, or Chinese celebrities and movies, teachers can design course content that includes more Chinese culture or celebrity-related teaching materials to enhance students’ interest in learning. Fourthly, this study only focuses on variables such as learners’ expectations and perceived quality. Future research could explore other dimensions that are of great research significance, such as learners’ self-efficacy and social presence. Lastly, while this study explores the learners’ individual differences, it does not incorporate them into the theoretical model. Future research could address this by including individual characteristics in the theoretical framework. By integrating factors such as learners’ demographics, age, language proficiency, weekly study duration, and prior learning experiences into the theoretical model, researchers can gain a deeper understanding of how these variables influence satisfaction levels. This would enable researchers to tailor interventions more effectively to enhance overall online learning satisfaction.

## Figures and Tables

**Table 1:** Participants’ information.

Item	Option	Frequency	Percentage (%)
Gender	Male	112	55.17
	Female	91	44.83
Country	Developed countries	94	46.31
	Developing countries	109	53.69
Age	Below 18 years old	27	13.30
	18–25 years old	60	29.56
	25–30 years old	83	40.89
	Above 30 years old	33	16.26
Major	Humanities and Social Sciences	84	41.38
	Science and Engineering	119	58.62
Chinese proficiency	Not participated or passed the HSK exam	15	7.39
	HSK level 1–2	77	37.93
	HSK level 3–4	76	37.44
	HSK level 5–6	35	17.24
Prior experience with offline Chinese courses	Yes	143	70.44
	No	60	29.56
Online course platform	Tencent meeting	45	22.17
	DingTalk	52	25.62
	Xuexi Tong	50	24.63
	Bilibili	34	16.75
	MOOC	17	8.37
	Other platforms	5	2.46
Weekly duration of online courses	More than 5 h	55	27.09
	3–5 h	76	37.44
	1–3 h	34	16.75
	Less than 1 h	38	18.72
Motivation for taking online courses	External motivation	93	45.81
	Internal motivation	110	54.19

**Table 2:** Statistical results for individual differences.

Variable	Gender		Countries		Age		Discipline		Chinese Proficiency Level		Experience with Offline Chinese Classes		Platforms		Study Duration		Motivation	
	*t*	*p*	*t*	*p*	*F*	*p*	*t*	*p*	*F*	*p*	*t*	*p*	*F*	*p*	*F*	*p*	*t*	*p*
Expectation	−0.321	0.749	1.692	0.092	1.495	0.217	1.136	0.257	1.243	0.295	−3.024	0.003	0.782	0.563	4.059	0.008	−1.143	0.255
Perceived Quality	−1.291	0.198	0.731	0.466	1.918	0.128	0.962	0.337	1.366	0.254	−3.827	0.000	2.797	0.018	4.802	0.003	0.130	0.897
Perceived Value	0.533	0.595	1.062	0.289	0.476	0.700	−0.387	0.699	2.011	0.114	−3.171	0.002	1.862	0.103	2.566	0.056	−0.189	0.850
Satisfaction	−1.165	0.245	−0.496	0.620	5.002	0.002	0.545	0.586	3.852	0.010	−10.776	0.000	2.074	0.070	19.638	0.000	0.359	0.720
Willingness to continue use	0.838	0.403	0.126	0.900	2.692	0.047	−0.614	0.540	1.452	0.229	−3.760	0.000	0.931	0.462	4.854	0.003	0.177	0.860

**Table 3:** Pearson correlation results for each variable.

Variable	Learner Expectation	Perceived Quality	Perceived Value	Learner Satisfaction	Willingness to Continue Use
Learner expectation	1				
Perceived quality	0.491 **	1			
Perceived value	0.525 **	0.455 **	1		
Learner satisfaction	0.533 **	0.570 **	0.528 **	1	
Willingness to continue use	0.501 **	0.512 **	0.510 **	0.530 **	1

Note: ** indicates a significant correlation at *p* < 0.01.

**Table 4:** Goodness-of-fit test results for the regression of each factor.

Model	Dependent Variable	Predictor Variables	*R*	*R* ^2^	Adjusted *R*^2^	Standard Error of Estimate	Durbin-Watson	*F*	*p*
Model 1	Perceived Quality	(Constant) learners’ expectations	0.491	0.241	0.237	0.92992	2.078	63.916	0.000
Model 1	Perceived Value	(Constant) learners’ expectations	0.525	0.276	0.272	0.74169		76.446	0.000
Model 2		(Constant) learner expectations, perceived quality	0.572	0.327	0.320	0.71680	2.112	48.524	0.000
Model 1	Satisfaction	(Constant) Perceived Quality	0.570	0.324	0.321	0.68685		96.544	0.000
Model 2		(Constant) Perceived Quality, Perceived Value	0.645	0.416	0.410	0.64041		71.131	0.000
Model 3		(Constant) Perceived Quality, Perceived Value, Learner Expectation	0.672	0.451	0.443	0.62218	1.908	54.540	0.000
Model 1	Willingness to Continue Using	(Constant) Learner Satisfaction	0.530	0.281	0.277	0.69664	1.815	78.395	0.000

**Table 5:** Regression coefficients for each factor.

Model	Dependent Variable	Predictor Variables	Unstandardized Coefficients		Standardized Coefficients	*t*	*p*	Collinearity Statistics	
			B	Std. Error	Beta			Tolerance	VIF
Model 1	Perceived quality	(Constant)	1.222	0.327		3.740	0.000		
		Learners’ expectations	0.623	0.078	0.491	7.995	0.000	1.000	1.000
Model 1	Perceived value	(Constant)	1.708	0.261		6.552	0.000		
		Learner expectations	0.543	0.062	0.525	8.743	0.000	1.000	1.000
Model 2		(Constant)	1.449	0.261		5.560	0.000		
		Learner expectations	0.411	0.069	0.397	5.965	0.000	0.759	1.318
		Perceived quality	0.212	0.054	0.260	3.899	0.000	0.759	1.318
Model 1	Learner Satisfaction	(Constant)	2.187	0.178		12.269	0.000		
		Perceived Quality	0.446	0.045	0.570	9.826	0.000	1.000	1.000
Model 2		(Constant)	1.363	0.222		6.128	0.000		
		Perceived Quality	0.325	0.048	0.415	6.844	0.000	0.793	1.261
		Perceived Value	0.325	0.058	0.339	5.586	0.000	0.793	1.261
Model 3		(Constant)	0.963	0.243		3.962	0.000		
		Perceived Quality	0.267	0.049	0.341	5.446	0.000	0.705	1.418
		Perceived Value	0.240	0.061	0.250	3.902	0.000	0.673	1.485
		Learner Expectation	0.233	0.065	0.235	3.591	0.000	0.644	1.552
Model 1	Willingness to continue using	(Constant)	1.781	0.233		7.646	0.000		
		Learner Satisfaction	0.521	0.059	0.530	8.854	0.000	1.000	1.000

**Table 6:** Statistics on preferred learning modes for Chinese language.

Item	Item Options	Frequency	Percentage (%)
Which mode do you prefer to learn Chinese	Offline mode	87	42.86
	Online mode	39	19.21
	Online and offline mixed mode	68	33.50
	No mode matters	9	4.43

## Data Availability

Data will be made available upon request.

## Supplementary Materials

The following supporting information can be downloaded at: https://www.mdpi.com/article/10.3390/bs14050387/s1, Supplementary S1: The American Customer Satisfaction Index (ACSI) model; Supplementary S2: The specific variables and questionnaire items; Supplementary S3: Online Chinese language course student satisfaction survey (Chinese and English Version); Supplementary S4: Basic information of interviewees; Supplementary S5: Results of Cronbach’s Alpha reliability test; Supplementary S6: KMO and Bartlett spherical inspection results; Supplementary S7: Specific statistical analysis of differences.
