# Supplementary material for: Factors Influencing the Satisfaction of Second Language Learners of Chinese in Online Courses
Source: Behav Sci (Basel). 2024 May 4;14(5):387. doi: 10.3390/bs14050387 (PMC11118836; doi:10.3390/bs14050387)
Supplement: Supplementary file 1 [file behavsci-14-00387-s001.zip › behavsci-2916237-supplementary.pdf]

Supporting Information for:

Factors Influencing the Satisfaction of Second Language Learners of Chinese in Online Courses

Supplementary S1. The American Customer Satisfaction Index (ACSI) model

Supplementary S2. The specific variables and questionnaire items

Supplementary S3. Online Chinese language course student satisfaction survey (Chinese and English Version)

Supplementary S4. Basic information of interviewees

Supplementary S5. Results of Cronbach's Alpha reliability test

Supplementary S6. KMO and Bartlett spherical inspection results

Supplementary S7. Specific statistical analysis of differences

Supplementary S1. The American Customer Satisfaction Index (ACSI) model

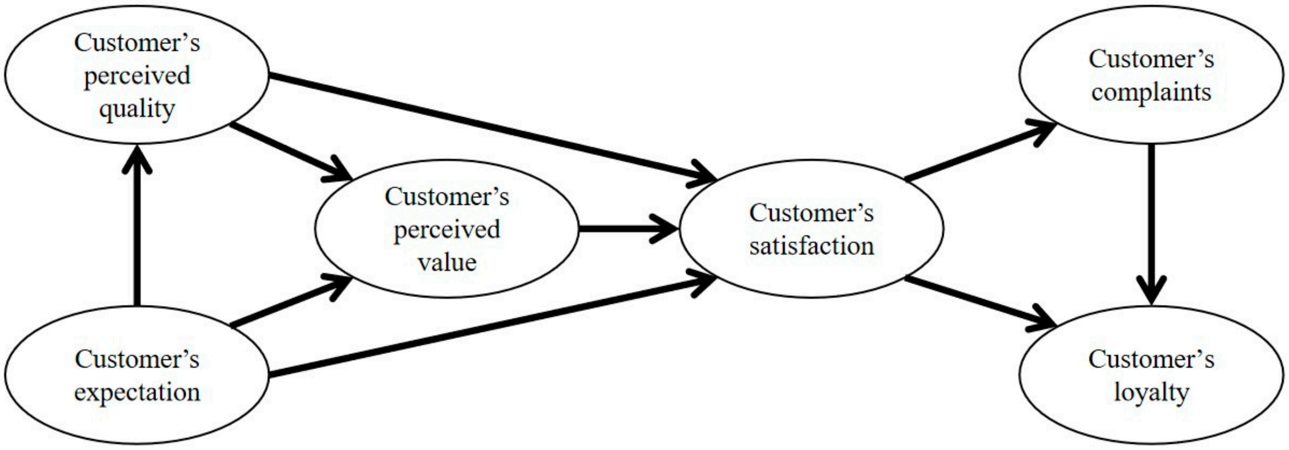

Figure S1 The American Customer Satisfaction Index (ACSI) model (Adapted from Fornell et al., (1996))

Supplementary S2. The specific variables and questionnaire items

Table S1 Variable Items

<table>
<tr><th>Latent Variables</th><th colspan="2">Observation Variable Items</th></tr>
<tr><td>Learner Expectations</td><td colspan="2">Expectations for online teaching content and format (Q1)
Expectations for the teacher's sense of responsibility and teaching level (Q2)
Expectations for improving oral and written skills through online teaching (Q3)
Expectations for online platform support services (Q4)</td></tr>
<tr><td rowspan="3">Perceived Quality</td><td>Quality of Teaching</td><td>Language used by the teacher for teaching (Q5)
Teaching methods and teaching content (Q6)
Communication and interaction between teachers and students (Q7)
Reasonableness of homework and exams (Q8)</td></tr>
<tr><td>Quality of Autonomous Learning</td><td>Lateness and absenteeism (Q9)
Preview and review situations (Q10)
Classroom performance (Q11)</td></tr>
<tr><td>Quality of the Network and Platform：</td><td>Platform functions (Q12)
Learning resources provided by online teaching (Q13)
Electronic devices used in class (Q14)
Network speed and stability (Q15)
Online course learning environment (Q16)</td></tr>
<tr><td>Perceived Value</td><td colspan="2">Improvement in learning efficiency by online courses (Q17)
Improvement in learning interest by online courses (Q18)
Whether there is motivation to explore problems (Q19)</td></tr>
<tr><td>Learner Satisfaction</td><td colspan="2">Gains from using online courses (Q20)
Whether the process of using online courses is pleasant (Q21)
Degree of satisfaction with the learning content (Q22)
Degree of satisfaction with the lecturer (Q23)
Degree of satisfaction with the platform used (Q24)</td></tr>
<tr><td>Willingness to Continue Using</td><td colspan="2">Whether willing to recommend online courses (Q25)
Whether will continue to choose the course in the future (Q26)
Whether willing to prioritize the lecturer's online course (Q27)
Whether willing to prioritize the online courses on this platform (Q28)</td></tr>
</table>

Supplementary S3. Online Chinese language course student satisfaction survey (Chinese and English Version)

亲爱的同学：

您好！为了深入了解影响线上对外汉语课程满意度的重要因素，以改善教学质量，提高学习者对线上教学的满意度，特进行此次问卷调查。您只需根据自身实际情况填写即可。本调查匿名进行，保证您信息的保密性。敬请您认真作答！非常感谢您的支持与配合！

您是否参加过线上对外汉语课程的学习？如果没有，请结束问卷的回答，感谢您的支持！

Dear students:

In order to deeply explore the important factors affecting the satisfaction of foreign students in online courses of teaching Chinese as a foreign language, and to improve the course quality and students' satisfaction, this questionnaire survey was conducted. You only need to fill it out according to your actual situation. This survey is conducted anonymously to ensure the confidentiality of your information. Please answer carefully! Thank you very much for your support and cooperation!

Have you ever attended an online Chinese course? If not, please finish to answer the questionnaire and thank you for your support!

第一部分 个人基本信息// Part I Personal Information

1. 您的国家是：// Your country is:_________

2. 您的性别是：// Your gender is:

①男 // Male ②女 // Female

3. 您的年龄是：// Your age is:

①18 岁以下 // under 18 years old

②18-25 岁 // 18-25 years old

③25-30 岁 // 25-30 years old

④30 岁以上 // over 30 years old

4. 您所学习的专业是：// Your major is:

①人文社科专业// Humanities and Social sciences

②理工专业//Science and Engineering

5. 您的汉语水平是：// Your Chinese proficiency is:

①未参加或通过 HSK 考试// haven’t passed HKS yet

②HSK 一-二级// HSK 1-2

③HSK 三-四级// HSK 3-4

④HSK 五-六级// HSK 5-6

6. 在进行线上课程之前，是否学过传统线下汉语课程？// Have you taken traditional Chinese courses before taking online courses?

①是// Yes ②否// No

7. 进行线上课程学习的平台是：// The platform for online courses is:

①腾讯会议// Tencent Conference

②钉钉// DingTalk

③学习通：//Learningpass

④哔哩哔哩// Bilibili

⑤慕课// MOOCS

⑥其他：// others:______

8. 每周进行线上课程学习时长为：// You learn online courses for______every week:

①5 小时以上// more than 5 hours

②3-5 小时// 3-5 hours

③1-3 小时// 1-3 hours

④1 小时以下// less than an hour

9. 进行线上课程的最主要目的是：// The most important purpose of taking online courses is to:

①提升自己的汉语知识水平和交际能力//enhance Chinese knowledge and communication skills

②对汉语言、文化或对中国的明星和影视感兴趣// be interested in Chinese language and culture or Chinese stars and movies

③希望多掌握一门外语，活到老学到老// master one more foreign language, and it’s never too late to learn

④求职需要// job hunting requires

⑤解决生活或学习中与他人的语言交流问题// solve the communication problems in life or study

⑥获得学历或学分// obtain academic qualifications or credits

⑦通过 HSK 水平考试//pass HSK examination

⑧证明自己的能力，得到他人或社会的认可// prove ability and secure the approbation of others or society.

⑨听从父母或他人的安排// obey the arrangement of parents or others

⑩其他：// other reasons:________

第二部分 线上课程满意度情况// Part II Satisfaction of online courses

学习者期望

1. 您对线上课程教学内容和形式的期望程度// Your expectation of the teaching content and form of online courses

①非常低// strongly low

②低// low

③一般// neutral

④高// high

⑤非常高// strongly high

2. 您对教师的责任心和教学水平的期望程度// Your expectation of teachers' sense of responsibility and teaching level

①非常低// strongly low

②低// low

③一般// neutral

④高// high

⑤非常高// strongly high

3. 您对线上课程能够提高您的汉语口语交际和读写能力的期望程度// Your expectation of online courses to improve your oral Chinese communication and reading and writing skills

①非常低// strongly low

②低// low

③一般// neutral

④高// high

⑤非常高// strongly high

4. 您对线上课程平台的支持服务（网络、设备、平台操作等）的期望程度// Your expectation of the support service (networking/facilities/platform function) of online courses

①非常低// strongly low

②低// low

③一般// neutral

④高// high

⑤非常高// strongly high

学习者感知质量（线上教学质量）

5. 教师的教学语言流畅清晰，通俗易懂// Teachers' teaching language is fluent, clear and easy to understand

①非常不认同// strongly disagree

②不认同// disagree

③一般 neutral

④认同// agree

⑤非常认同// strongly agree

6. 教师的教学方法生动多样，活动新颖丰富// Teachers' teaching methods are vivid and diverse, and the activities are novel and abundant

①非常不认同// strongly disagree

②不认同// disagree

③一般 neutral

④认同// agree

⑤非常认同// strongly agree

7. 教师经常与同学们进行交流互动// Teachers often interact with students

①非常不认同// strongly disagree

②不认同// disagree

③一般 neutral

④认同// agree

⑤非常认同// strongly agree

8. 作业和考试题型合理，难度适中，评分客观// The homework and exam questions are reasonable, the difficulty is moderate, and the score is objective

①非常不认同// strongly disagree

②不认同// disagree

③一般 neutral

④认同// agree

⑤非常认同// strongly agree

学习者感知质量（自主学习质量）

9. 您从不迟到或旷课// You are never late or absent from class

①非常不认同// strongly disagree

②不认同// disagree

③一般 neutral

④认同// agree

⑤非常认同// strongly agree

10. 您能做到课前预习，课后复习并完成作业// You can preview before class, review after class and finish your homework

①非常不认同// strongly disagree

②不认同// disagree

③一般 neutral

④认同// agree

⑤非常认同// strongly agree

11. 在课堂上，您愿意积极思考，主动发言，与同学和老师进行交流互动//In online class, you are willing to actively think and take the initiative to speak, and actively communicate with the classmates and teachers

①非常不认同// strongly disagree

②不认同// disagree

③一般 neutral

④认同// agree

⑤非常认同// strongly agree

学习者感知质量（网络和平台质量）

12. 平台功能全面、易于操作、稳定性程度高// The platform has comprehensive functions, easy operation and high stability

①非常不认同// strongly disagree

②不认同// disagree

③一般 neutral

④认同// agree

⑤非常认同// strongly agree

13. 线上课程提供的配套电子资源或教材能满足学习需求// Electronic resources or teaching materials provided by the course can meet your need

①非常不认同// strongly disagree

②不认同// disagree

③一般 neutral

④认同// agree

⑤非常认同// strongly agree

14. 课堂使用的电脑、麦克风和投影仪的呈现效果非常好// Presentation effect of computer, microphone and projector used in online classroom can be great

①非常不认同// strongly disagree

②不认同// disagree

③一般 neutral

④认同// agree

⑤非常认同// strongly agree

15. 您的网络速度和稳定度非常好// Your network speed and stability is great

①非常不认同// strongly disagree

②不认同// disagree

③一般 neutral

④认同// agree

⑤非常认同// strongly agree

16. 您的线上课程学习环境安静、利于线上学习// The environment of your online courses is quiet and conducive to e-learning

①非常不认同// strongly disagree

②不认同// disagree

③一般 neutral

④认同// agree

⑤非常认同// strongly agree

学习者感知价值

17. 线上课程提高了您的汉语学习效率和效果// Online Chinese courses have improved your efficiency and effectiveness

①非常不认同// strongly disagree
②不认同// disagree
③一般 neutral
④认同// agree
⑤非常认同// strongly agree

18. 线上课程提高了您的汉语学习兴趣// Online courses have enhanced your interest in Chinese learning

①非常不认同// strongly disagree
②不认同// disagree
③一般 neutral
④认同// agree
⑤非常认同// strongly agree

19. 您有动力也愿意花时间去探索解决课程中发现的问题// You are motivated and willing to explore and solve the problems found in the course

①非常不认同// strongly disagree
②不认同// disagree
③一般 neutral
④认同// agree
⑤非常认同// strongly agree

学习者满意度

20. 线上课程使您收获很大// Online courses have made you gain a lot

①非常不认同// strongly disagree
②不认同// disagree

③一般 neutral

④认同// agree

⑤非常认同// strongly agree

21. 使用线上课程的实际过程令人愉快// The actual process of using online courses is enjoyable.

①非常不认同// strongly disagree

②不认同// disagree

③一般 neutral

④认同// agree

⑤非常认同// strongly agree

22. 您对线上课程学习内容的满意程度// Your satisfaction with the content of online courses

①非常不满意// strongly dissatisfied

②不满意// dissatisfied

③一般// neutral

④满意// satisfied

⑤非常满意// strongly satisfied

23. 您对主讲教师的满意程度// Your satisfaction with the lecturer

①非常不满意// strongly dissatisfied

②不满意// dissatisfied

③一般// neutral

④满意// satisfied

⑤非常满意// strongly satisfied

24. 您对所使用教学平台的满意程度// Your satisfaction with the platform

①非常不满意// strongly dissatisfied

②不满意// dissatisfied

③一般// neutral

④满意// satisfied

⑤非常满意// strongly satisfied

持续使用意愿

25. 您愿意把线上课程推荐给别人// You are willing to recommend online courses to others

①非常不认同// strongly disagree

②不认同// disagree

③一般 neutral

④认同// agree

⑤非常认同// strongly agree

26. 您愿意未来继续使用线上汉语课程// You are willing to continue to use online Chinese courses in the future

①非常不认同// strongly disagree

②不认同// disagree

③一般 neutral

④认同// agree

⑤非常认同// strongly agree

27. 如果要学习新的知识，您会优先选择该教师的线上课程// If you need to learn new knowledge, you prefer the lecturer's online courses

①非常不认同// strongly disagree

②不认同// disagree

③一般 neutral

④认同// agree

⑤非常认同// strongly agree

28. 如果要学习新的知识，您会优先选择该平台的线上课程// If you need to learn new knowledge, you prefer the platform for online courses

①非常不认同// strongly disagree

②不认同// disagree

③一般 neutral

④认同// agree

⑤非常认同// strongly agree

线上线下对比

29. 更喜欢哪种模式来学习汉语// Which way do you prefer to learn Chinese?

①线下教学// traditional courses

②线上教学// online courses

③线上和线下混合教学// combination of online and traditional courses

④任何模式都无所谓// It doesn’t matter

Supplementary S4. Basic information of interviewees

Table S2 Basic Information of Interviewees

Interviewee	Gender	Chinese Proficiency	Country
A	Male	HSK5	Japan
B	Female	HSK4	Russia
C	Female	HSK4	New Zealand
D	Male	HSK4	Thailand
E	Female	HSK3	Australia

Supplementary S5. Results of Cronbach's Alpha reliability test

Table S3 Results of Cronbach's Alpha reliability test

Variables	Cronbach's Alpha	Number of items
Learner expectations	0.852	4
Perceived quality	0.940	12
Perceived value	0.802	3
Learner satisfaction	0.839	5
Willingness to continue using	0.853	4
Overall reliability	0.947	28

Supplementary S6. KMO and Bartlett spherical inspection results

Table S4 KMO and Bartlett spherical inspection results

KMO sampling suitability quantity		0.938
Bartlett spherical test	Approximate chi square	3299.873
	Degrees of Freedom	378
	p	0.000

Supplementary S7. Specific statistical analysis of differences

4.2.1 Analysis of gender differences

An independent sample *t*-test was used to examine gender differences. Table S5 shows that the *p*-values for gender differences in the five dimensions of learner expectations, perceived quality, perceived value, learner satisfaction, and intention to continue using are all greater than 0.05. This indicates that gender differences do not have a significant impact on learner satisfaction in online Chinese language teaching.

Table S5 Independent sample *t*-tests for gender on various variables

Variable	Gender	Sample Size	Mean	Standard Deviation	*t*	*p*
Expectation	Male	112	4.09	0.888	-0.321	0.749
	Female	91	4.13	0.781		
Perceived Quality	Male	112	3.70	1.143	-1.291	0.198
	Female	91	3.89	0.957		
Perceived Value	Male	112	3.97	0.933	0.533	0.595
	Female	91	3.90	0.788		
Satisfaction	Male	112	3.81	0.847	-1.165	0.245
	Female	91	3.95	0.815		
Intention to Continue Use	Male	112	3.84	0.810	0.838	0.403

4.2.2 Analysis of differences among countries

To analyze the differences among countries, the authors divided them into two categories: developed countries and developing countries, and performed an independent sample *t*-test. Table S6 shows the results of the differences in each variable between the two categories.

Table S6 Analysis of variance for different countries on various variables

Variable	Country	Sample Size	Mea n	Standard Deviation	*t*	*p*

Expectation	Developed Countries	94	4.22	0.692	1.692	0.092
	Developing Countries	109	4.02	0.943		
Perceived Quality	Developed Countries	94	3.84	1.043	0.731	0.466
	Developing Countries	109	3.73	1.085		
Perceived Value	Developed Countries	94	4.01	0.798	1.062	0.289
	Developing Countries	109	3.88	0.926		
Satisfaction	Developed Countries	94	3.84	0.813	- 0.496	0.620
	Developing Countries	109	3.90	0.854		
Intention to Continue Use	Developed Countries	94	3.81	0.772	0.126	0.900
	Developing Countries	109	3.79	0.862		

As indicated in Table S6, neither developed countries nor developing countries reached a significant level of 0.05 in the significance test for each dimension. That is, the *p*-values were all greater than 0.05, suggesting that there was no significant difference among learners from different countries in terms of learner expectations, perceived quality, perceived value, satisfaction, and intention to continue using the online course. This finding may be because the conditions for learning Chinese online are not particularly high. Both developed and developing countries have the necessary conditions for learning Chinese, including network systems and platform support.

4.2.3 Analysis of different age groups

To investigate the impact of age, a one-way analysis of variance was conducted to test for differences among age groups. As shown in Table S7 the *p*-values for age in the dimensions of learner expectations, perceived quality, and perceived value were greater than 0.05, indicating that there were no significant differences in these three dimensions among different age groups. However, the *p*-values for age in satisfaction and willingness to continue using were less than 0.05, suggesting that significant differences existed in learner satisfaction and willingness to continue using among different age groups.

The mean satisfaction score for learners under 18 was 3.39, while the mean scores for learners aged 18-25, 25-30, and over 30 were 3.79, 4.04, and 4.02, respectively. Post-hoc tests using LSD revealed that there were significant differences in satisfaction between learners under 18 and the other three age groups, with satisfaction scores in the following order: under 18 < 18-25 < 25-30 < over 30. In terms of willingness to continue using, the mean score for learners under 18 was 3.43, while the mean scores for learners aged 18-25, 25-30, and over 30 were 3.79, 3.93, and 3.77, respectively. Post-hoc tests using LSD indicated that there was a significant difference in willingness to continue using between learners under 18 and those aged 25-30, with willingness scores in the following order: under 18 < 25-30.

Table S7 presents the results of the One-way Analysis of Variance (ANOVA) analysis of the variables by age group. The variables include expectation, perceived quality, perceived value, satisfaction, and intention to continue using the online course for teaching Chinese as a foreign language.

Table S7 ANOVA result for age on each variable

Variable	Age Range	Sample Size	Mean	Standard Deviation	*F*	*p*	LSD
Expectation	Below 18	27	3.93	1.213	1.495	0.217	
	18-25	60	4.10	0.809			
	25-30	83	4.24	0.719			
	30 and above	33	3.95	0.792			
Perceived Quality	Below 18	27	3.51	1.199	1.918	0.128	
	18-25	60	3.70	1.116			
	25-30	83	3.98	0.942			
	30 and above	33	3.65	1.105			
Perceived Value	Below 18	27	3.88	1.030	0.47	0.70	

	18-25	60	3.91	0.876	6	0	
	25-30	83	4.02	0.758			
	30 and above	33	3.84	0.993			
Satisfaction	Below 18	27	3.39	0.640	5.002	0.002	1＜2，3，4
	18-25	60	3.79	0.843			
	25-30	83	4.04	0.725			
	30 and above	33	4.02	1.043			
Intention to Continue Using	Below 18	27	3.43	0.820	2.693	0.047	1＜3
	18-25	60	3.79	0.938			
	25-30	83	3.93	0.740			
	30 and above	33	3.77	0.708			

Note: In the post hoc LSD test, below 18 is labeled as 1; 18-25 is labeled as 2; 25-30 is labeled as 3; 30 and above is labeled as 4.

The results show that there are significant differences in satisfaction and intention to continue using the online course across different age groups. Specifically, learners below 18 years old have lower satisfaction and intention to continue using the online course than other age groups. Learners' satisfaction increases as their age increases. The possible reasons for this phenomenon could be related to their learning experiences and achievement goals. Younger learners may feel more pressure from school requirements and academic performance, while older learners may have more flexible learning plans and goals.

According to the average values, learner satisfaction increases with age. This may be due to several reasons. Firstly, it could be related to the learners' educational background. Students in different age

groups have varied educational experiences, with most learners under 18 and between 18-25 being students in school, for whom online Chinese courses are either compulsory or elective courses. On the other hand, learners over 25 are mostly working professionals for whom online Chinese courses serve as supplementary courses in their spare time. Compared to extracurricular tutoring courses, school courses are more difficult, require credits, and are more important. Younger learners experience more pressure from online learning, which may lead to lower satisfaction and, therefore, lower intention to continue using these courses compared to older learners. Secondly, it may be related to learners' achievement goals. Achievement goals can be divided into mastery goals and performance goals (Sarwar et al. 2009), with mastery goals focusing on knowledge acquisition and performance goals focusing on self-evaluation. Middle and high school students are more influenced by mastery goals and place greater emphasis on mastering knowledge, attaching importance to teacher-student interactions, and hoping for stricter supervision from teachers, thus resulting in lower satisfaction with online courses than older learners. On the other hand, online courses offer greater flexibility in learning and allow for the development of a personalized, flexible learning plan, making them more suitable for older working professionals, resulting in higher satisfaction with online courses among this group.

4.2.4 Analysis of differences between different majors

To examine the differences in learner satisfaction across different academic disciplines, independent samples *t*-tests were conducted. Table S8 presents the results of the independent samples t-tests for each variable across the two academic disciplines.

Table S8 Independent samples *t*-test for variables across different academic disciplines

Variable	Academic Discipline	Sample Size	Mean	Standard Deviation	*t*	*p*
Expectancy	Humanities and Social Sciences	84	4.19	0.763	1.136	0.257
	Science and Engineering	119	4.05	0.889		
Perceived Quality	Humanities and Social Sciences	84	3.87	0.974	0.962	0.337

	Science and Engineering	119	3.72	1.125		
Perceived Value	Humanities and Social Sciences	84	3.91	0.785	-0.387	0.699
	Science and Engineering	119	3.96	0.927		
Satisfaction	Humanities and Social Sciences	84	3.91	0.766	0.545	0.586
	Science and Engineering	119	3.85	0.880		
Continued Use Intention	Humanities and Social Sciences	84	3.76	0.820	-0.614	0.540
	Science and Engineering	119	3.83	0.821		

To investigate the differences in learner satisfaction between different academic disciplines, the independent samples *t*-test method was used. As shown in Table S8, the *p*-values for both academic disciplines were greater than 0.05 across all five dimensions, indicating that there were no significant differences in learner satisfaction between the two academic disciplines. This may because the partiipants came from comprehensive universities where there were no major differences in the language courses and teaching faculty between the humanities and social sciences and science and engineering programs.

4.2.5 Analysis between different Chinese proficiency levels

Table S9 shows the results of the one-way ANOVA analysis of each variable for different Chinese proficiency levels.

Table S9 the one-way ANOVA analysis of each variable for different Chinese proficiency levels

Variable	Chinese Proficiency Level	Sample Size	Mean	Standard Deviation	F	*p*	LSD
Expectations	Not taken or passed HSK	15	3.83	0.869	1.243	0.295	

	HSK1-2	77	4.23	0.666			
	HSK3-4	76	4.09	0.937			
	HSK5-6	35	4.01	0.935			
Perceived Quality	Not taken or passed HSK	15	3.35	1.389	1.366	0.254	
	HSK1-2	77	3.83	0.966			
	HSK3-4	76	3.89	1.101			
	HSK5-6	35	3.63	1.025			
Perceived Value	Not taken or passed HSK	15	3.62	1.112	2.011	0.114	
	HSK1-2	77	4.04	0.797			
	HSK3-4	76	3.82	0.870			
	HSK5-6	35	4.11	0.871			
Satisfaction	Not taken or passed	15	3.39	0.857	3.852	0.010	1＜3，4；

	HSK1-2	77	3.77	0.617			2<4
	HSK3-4	76	3.93	0.935			
	HSK5-6	35	4.17	0.905			
Intention to Continue Using	Not taken or passed	15	3.42	1.305	1.452	0.229	
	HSK1-2	77	3.89	0.680			
	HSK3-4	76	3.79	0.808			
	HSK5-6	35	3.77	0.852			

Note: In the post-hoc test LSD, not taken or passed HSK is labeled as 1, HSK1-2 as 2, HSK3-4 as 3, and HSK5-6 as 4.

Table S9 reveals that the *p*-values for Chinese language proficiency in terms of learner expectations, perceived quality, perceived value, and intention to continue using the course are all greater than 0.05, indicating that there are no significant differences. However, in terms of learner satisfaction (*p*=0.010), indicating that there is a significant difference in satisfaction levels among learners with different Chinese language proficiencies. The average satisfaction levels for learners who did not pass the HSK exam is 3.39, for HSK1-2 level learners it is 3.77, for HSK3-4 level learners it is 3.93, and for HSK5-6 level learners it is 4.17. It can be observed that the average satisfaction level increases gradually with increasing Chinese language proficiency. The post-hoc LSD test reveals that the satisfaction level of learners who did not pass the HSK exam is significantly lower than that of learners at the HSK3-4 and HSK5-6 levels, while the satisfaction level of HSK1-2 level learners is significantly lower than that of

HSK5-6 level learners. This may because learners who did not pass the Chinese language proficiency test and HSK1-2 level learners are beginners or have a lower level of proficiency in Chinese, and therefore have a slower learning pace and lower learning efficiency in the classroom compared to learners at a higher level. As a result, their satisfaction level is lower compared to those of higher-level students. On the other hand, intermediate and advanced-level Chinese learners have already acquired a certain level of Chinese knowledge and foundation, and may be more adept at online courses, which can lead to higher learning progress and more positive learning outcomes. Therefore, it can be concluded that intermediate and advanced-level Chinese learners have a higher level of satisfaction compared to beginners or low-level Chinese learners.

4.2.6 Verification of prior experience with offline Chinese courses

This section presents the results of the independent sample *t*-test conducted to examine the difference in prior experience with offline Chinese courses. Table S10 shows the independent sample *t*-test for each variable of whether the learners have learned offline Chinese courses.

Table S10 Independent Samples *t*-test for variables on whether attended offline Chinese courses

Variable	Option	Sample Size	Mean	Standard Deviation	*t*	*p*
Expectation	Yes	143	4.02	0.922	-3.024	0.003
	No	60	4.33	0.546		
Perceived Quality	Yes	143	3.64	1.179	-3.827	0.000
	No	60	4.12	0.608		
Perceived Value	Yes	143	3.84	0.960	-3.171	0.002
	No	60	4.18	0.537		
Satisfaction	Yes	143	3.59	0.798	-10.776	0.000
	No	60	4.54	0.447		

Intention to Continue Using	Yes	143	3.69	0.909	-3.760	0.000
	No	60	4.05	0.463		

An independent sample *t*-test was conducted to investigate the impact of whether learners had previous offline Chinese language learning experience on their satisfaction with online courses. Table 10 presents the results of the *t*-test for each variable. The *p*-values for learner expectations, perceived quality, perceived value, learner satisfaction, and intention to continue using online courses were all found to be less than 0.05, indicating significant differences between learners who had previous offline learning experience and those who did not, across all five dimensions.

The average values for each dimension show that learners who had previous offline learning experience had significantly lower scores than those who did not. Specifically, the mean satisfaction score for learners who had not studied offline was 4.54, while the mean satisfaction score for those who had was 3.59. These findings suggest that learners with prior offline learning experience may have a preference for traditional classroom learning or may find it difficult to adapt to online learning, leading to lower satisfaction with online courses. Overall, these results highlight the importance of considering learners' previous learning experiences when designing and implementing online language courses to enhance learner satisfaction.

4.2.7 Verification of differences between different platforms

This section reports the results of the one-way ANOVA test to examine the difference among different platforms. Table S11 displays the mean, standard deviation, F-value, *p*-value, and LSD value for each dimension of the different platforms. The dimensions include expectation, perceived quality, perceived value, satisfaction, and intention to continue using.

Table S11 ANOVA for different platforms on each dimension

Dimension	Platform	Sample Size	Mean	Standard Deviation	*F*	*p*	LSD
Expectation	Tencent Meeting	45	4.06	0.940	0.782	0.563	
	DingTalk	52	4.18	0.785			
	Chaoxing Learning Pass	50	3.99	0.862			
	Bilibili	34	4.3	0.761			

			0				
	MOOC	17	4.10	0.718			
	ZOOM	5	3.85	1.194			
Perceived Quality	Tencent Meeting	45	3.75	1.062	2.797	0.018	3＜2，4，5
	DingTalk	52	3.98	1.015			
	Chaoxing Learning Pass	50	3.37	1.311			
	Bilibili	34	4.02	0.685			
	MOOC	17	4.13	0.703			
	ZOOM	5	3.40	1.018			
Perceived Value	Tencent Meeting	45	3.92	0.856	1.862	0.103	
	DingTalk	52	4.05	0.793			
	Chaoxing Learning Pass	50	3.75	1.070			

	Bilibili	34	3.8<br>2	0.766			
	MOOC	17	4.2<br>9	0.564			
	ZOOM	5	4.5<br>3	0.447			
Satisfaction	Tencent Meeting	45	3.7<br>2	0.858	2.0<br>74	0.0<br>70	
	DingTalk	52	3.9<br>0	0.741			
	Chaoxing Learning Pass	50	3.7<br>2	0.984			
	Bilibili	34	4.0<br>8	0.659			
	MOOC	17	4.2<br>9	0.490			
	ZOOM	5	3.6<br>0	1.349			
Intention to Continue Using	Tencent Meeting	45	3.8<br>0	0.795	0.9<br>31	0.4<br>62	
	DingTalk	52	3.8<br>7	0.779			
	Chaoxing Learning Pass	50	3.6<br>4	1.017			

	Bilibili	34	3.95	0.536			
	MOOC	17	3.87	0.786			
	ZOOM	5	3.40	0.962			

Note: In the post hoc LSD test, Tencent Meeting is labeled as 1; DingTalk is labeled as 2; Tencent Classroom is labeled as 3; Bilibili is labeled as 4; MOOC is labeled as 5; ZOOM is labeled as 6.

Table S11 reveals that different platforms have a significant difference in perceived quality (p=0.018). However, the p-values for the other four dimensions are greater than 0.05, indicating no significant difference among platforms in these dimensions. On the dimension of perceived quality, the average score for Tencent Meeting is 3.75, DingTalk is 3.98, StudyTube is 3.37, Bilibili is 4.02, MOOC is 4.13, and ZOOM is 3.40. The post hoc LSD test shows that learners who used StudyTube have significantly lower perceived quality scores than those who used DingTalk, Bilibili, and MOOC. The possible reason for this result may be that the learners using StudyTube were high school or university students, and StudyTube was a mandatory software required by the school, not their own choice. Therefore, they may not adapt well, leading to unsatisfactory learning outcomes. Consequently, the learners' satisfaction scores are not high compared to other platforms. Different online learning platforms have no significant difference in all dimensions except for perceived quality.

4.2.8 Test for differences in learning duration

ANOVA analysis and a post-hoc LSD test was conducted to examine differences in perceived quality, perceived value, satisfaction, and willingness to continue using the course based on the weekly study time of learners.

Table S12 ANOVA results for study duration on each variable

Variable	Duration	Sample Size	Mean	Standard Deviation	F	p	LSD

Expectation	More than 5 hours	55	3.80	1.096	4.059	0.008	1＜2，3，4
	More than 5 hours	76	4.16	0.776			
	1-3 hours	34	4.33	0.586			
	Less than 1 hour	38	4.26	0.590			
Perceived Quality	More than 5 hours	55	3.37	1.309	4.802	0.003	1＜2，3，4
	More than 5 hours	76	3.82	0.985			
	1-3 hours	34	3.96	0.731			
	Less than 1 hour	38	4.14	0.912			
Perceived Value	More than 5 hours	55	3.67	1.065	2.566	0.056	
	More than 5 hours	76	4.00	0.809			
	1-3 hours	34	4.09	0.683			
	Less than 1 hour	38	4.08	0.757			

Satisfaction	More than 5 hours	55	3.30	0.766	19.638	0.000	1＜2，3，4；2＜4
	More than 5 hours	76	3.88	0.687			
	1-3 hours	34	4.18	0.735			
	Less than 1 hour	38	4.41	0.796			
Intention to Continue Using	More than 5 hours	55	3.45	1.089	4.854	0.003	1＜2，3，4
	More than 5 hours	76	3.94	0.709			
	1-3 hours	34	3.95	0.557			
	Less than 1 hour	38	3.88	0.623			

Note: In the post hoc LSD test, “More than 5 hours” is labeled as 1; “3-5 hours” is labeled as 2; “1-3 hours” is labeled as 3; “Less than 1 hour” is labeled as 4.

The results of the ANOVA showed that there was no significant difference in perceived value among learners with different weekly study times (p=0.056). However, there were significant differences in learners’ expectations, perceived quality, satisfaction, and willingness to continue using the course among learners with different weekly study times (p<0.05). The LSD post-hoc test showed that learners who studied for five or more hours per week had significantly lower scores in expectations, perceived quality, satisfaction, and willingness to continue using the course compared to learners in other groups. Additionally, the satisfaction scores of learners who studied for 3-5 hours per week were significantly lower than those who studied for less than one hour per week.

Most learners who study for more than five hours per week may perceive online learning as a mandatory course, and may experience fatigue due to the long duration of daily learning. This may lead to a lower evaluation of satisfaction with online learning.

4.2.9 Differential analysis of learning motivation

Table S13 reports the results of the independent sample t-test on the variables of learning motivation.

Table S13 Independent samples *t*-test for study motivation variables.

Variable	Motivation	Sample Size	Mean	Standard Deviation	*t*	*p*
Expectation	Internal Motivation	93	4.04	0.929	-1.143	0.255
	External Motivation	110	4.17	0.756		
Perceived Quality	Internal Motivation	93	3.79	1.061	0.130	0.897
	External Motivation	110	3.77	1.073		
Perceived Value	Internal Motivation	93	3.93	0.909	-0.189	0.850
	External Motivation	110	3.95	0.838		
Satisfaction	Internal Motivation	93	3.90	0.850	0.359	0.720
	External Motivation	110	3.85	0.823		

Intention to Continue Using	Internal Motivation	93	3.81	0.782	0.177	0.860
	External Motivation	110	3.79	0.853		

In the items on learning motivation, the questionnaire contained 9 options, including “improving one’s knowledge of Chinese and communication skills,” “interest in Chinese language, culture, or Chinese celebrities and films,” “hoping to master one more foreign language and keep learning,” “job requirement,” “solving language communication problems in life or study with others,” “obtaining a degree or elective credits,” “taking the HSK level exam,” “proving one’s ability and gaining recognition from others or society,” and “following parents’ or others’ arrangements.” Participants were required to select one option as their main motivation for learning Chinese online, i.e., learning motivation. The first three options are internal motivation, and the following six are external motivation. In this study, learning motivation was divided into internal and external motivation, and the independent sample *t*-test was used for differential analysis. As shown in Table S13, the significance of internal and external motivation in all dimensions is greater than 0.05, indicating that there is no significant difference in learner expectations, perceived quality, perceived value, satisfaction, and intention to continue using among participants with different learning motivations.
